# Comparative effects of high-intensity interval training versus moderate-intensity continuous training on body composition and blood pressure in overweight adolescents: a systematic review and meta-analysis of randomized controlled trials

**DOI:** 10.3389/fphys.2025.1636792

**Published:** 2025-10-01

**Authors:** Xilin Li, Zhibo Cui, Zhijun Tan, Jiangxuan Li, Chengbo Yang

**Affiliations:** School of Sport Training, Chengdu Sport University, Chengdu, China

**Keywords:** HIIT, MICT, overweight adolescents, body composition, meta-analysis

## Abstract

**Background and aims:**

Adolescent overweight and obesity are increasing worldwide, posing a growing public health concern. Although both HIIT and MICT have been shown to reduce body fat and improve cardiovascular fitness, few studies have directly compared their effects in adolescents. This study therefore systematically evaluates their impact on body composition and blood pressure to inform appropriate exercise recommendations.

**Methods:**

An extensive database search was undertaken involving six sources—such as PubMed, Web of Science, and the Cochrane Library—employing predetermined search terms to locate randomized controlled trials. The search covered literature published up to February 20, 2025, starting from each database’s inception. The methodological rigor of the selected studies was evaluated through a revised evaluation approach based on PRISMA guidelines. Finally, the influence of the two exercise modalities on adiposity reduction and blood pressure in overweight/obese adolescents was analyzed and discussed.

**Results:**

An overall number of 16 randomized controlled trials (RCTs), comprising 473 eligible participants, were incorporated into the analysis. The findings revealed that: (1) HIIT was superior in decreasing body fat percentage [MD = −0.74, 95% CI (−1.44, 0.04), Z = 2.74, P = 0,04]. There was no statistically relevant difference detected between the two groups in body weight, body mass index, fat-free mass, or blood pressure. (2) Subgroup analysis indicated that MICT was more effective in reducing waist circumference when running was used as the mode of exercise intervention [MD = 2.81, 95% CI (0.36, 5.26), Z = 2.25, P = 0.02].

**Conclusion:**

Both HIIT and MICT demonstrated comparable effects on most outcomes in overweight/obese adolescents. HIIT showed a modest, borderline significant advantage in reducing body fat percentage, while MICT appeared more effective in reducing waist circumference in the running subgroup. Further high-quality studies are needed to confirm these findings and guide exercise recommendations.

**Systematic Review Registration:**

https://www.crd.york.ac.uk/PROSPERO/#myprospero, identifier CRD42025639229.

## 1 Introduction

Adolescent obesity has emerged as an increasingly serious global public health issue. As reported by the World Health Organization (WHO), the year 2022 saw over 390 million individuals aged 5 to 19 classified as overweight, with approximately 160 million falling into the obese category. The global prevalence of overweight among this age group increased fourfold, from 2% to 8%, compared to data from 2019 (World Health Organization, 2024a). Obesity has become one of the most serious global epidemics and is characterized by excessive fat accumulation (World Health Organization, 2021). WHO identifies obesity as a complex condition with multiple contributing factors, and it is now officially listed as a disease in the International Classification of Diseases, 11th Revision (ICD-11) (World Health Organization, 2022). Obesity-related complications may have long-term impacts on adolescents, including metabolic disorders, impaired sleep quality, reduced physical activity, and hindered growth and development. More seriously, it may impair bone health and reproductive function, and significantly increase the potential for type 2 diabetes onset, cardiovascular conditions, specific types of cancer (World Health Organization, 2019; Skinner et al., 2015), and even premature mortality (Freedman et al., 2007).

Obesity is currently recognized as stemming from a disparity in energy intake and expenditure (Bray et al., 2017). Reducing fat and body weight through dieting or energy-restrictive methods in overweight or obese adolescents is likely to result in potential health risks, such as malnutrition, decreased basal metabolic rate, and impaired growth. Exercise, as a non-pharmacological intervention, is widely considered an effective approach for reducing fat and weight in adolescents. WHO findings indicate that over 80% of school-aged youth worldwide fail to reach the recommended physical activity recommendation of at least 1 h daily (World Health Organization, 2019). As per the American College of Sports Medicine (ACSM)’s documented guidelines on evaluating as well as prescribing exercise, it is indicated that MICT is a traditional and well-supported intervention suitable for a broad population, including individuals with obesity (ACoS, 2018). With increasing research attention, HIIT has emerged as a time-efficient training modality and has been progressively applied to individuals with various health conditions. When professionally supervised, HIIT can also be safely implemented in adolescents and individuals with obesity. However, careful adjustments are required regarding exercise intensity, recovery periods, and individual responsiveness. The 2020 statement issued by the European Association of Preventive Cardiology (EAPC) highlighted HIIT as a valuable approach in cardiovascular rehabilitation and metabolic disease intervention, demonstrating favorable effects on significant health markers such as maximal oxygen uptake, blood pressure, blood glucose, and body fat. The statement further emphasizes that HIIT and MICT should be flexibly tailored based on individual fitness levels, health status, and adherence capacity, with special attention to adaptability and long-term sustainability in specific populations, such as adolescents (Hansen et al., 2017).

Body mass and composition serve as key metrics in assessing the impact of exercise interventions among overweight or obese populations (ACoS, 2018). Achieving sustained health benefits requires not only reducing excess adiposity but also maintaining or enhancing lean muscle mass (Stefanakis et al., 2024). Blood pressure plays a critical role in the progression of hypertension and cardiac metabolic regulation, and its regulation is crucial for preventing cardiovascular complications in overweight/obese youths (Urbina et al., 2023). There is growing evidence that high-intensity interval training (HIIT) and moderate-intensity continuous training (MICT) may exert differential impact on vascular performance and autonomic nervous control (Su et al., 2024). Metabolic changes are closely associated with weight gain and elevated blood pressure (Shi et al., 2022).

The influence of HIIT and MICT on weight management and cardiovascular metabolic health has been extensively studied (Zheng et al., 2025). Nevertheless, the relative effectiveness of these two training approaches continues to be a topic of ongoing debate. MICT is commonly used to enhance physical activity levels (World Health Organization, 2020). Due to its moderate intensity, it is considered suitable for beginners or individuals with limited physical conditioning, and could lower the likelihood of sustaining exercise-related injuries (Baltich et al., 2017). As a result, MICT has been widely adopted to promote general physical health. A recent meta-analysis found that MICT gave rise to a notable decrease in body fat percentage (BF%) in overweight/obese adults, although subgroup analysis indicated that HIIT could lead to a more substantial reduction in BF% than MICT (Poon et al., 2024), with no notable differences found between the two in other outcomes (Keating et al., 2017). However, the suitability of MICT for adolescents aged 10–19 remains controversial. Adolescents tend to engage in spontaneous and high-intensity physical activities under natural conditions (Bailey et al., 1995). HIIT consists of brief bursts of high-intensity activity interspersed with periods of rest or low-intensity exercise, leading to substantial physiological changes in a relatively short amount of time, thereby enhancing cardiovascular health, reducing fat mass, and improving metabolic function (Baquet et al., 2017). Over the past few years, HIIT has increasingly been adopted as a common exercise intervention for obese adolescents.

The relative effectiveness of HIIT and MICT for overweight/obese adolescents aged 10–19 warrants further investigation, as previous studies have been limited by small sample sizes, short intervention durations, or lack of direct comparisons between the two modalities. By conducting a comprehensive review and quantitative synthesis, the comparative impact of HIIT and MICT on body mass, body composition, and blood pressure in overweight/obese adolescents will be examined, aiming to offer evidence-based guidelines for exercise prescriptions in obesity management and inform future clinical decision-making.

## 2 Methods

This study was conducted in conformity with the PRISMA Statement for systematic reviews and meta-analyses. And it adhered to the PERSiST Guidelines (Page et al., 2021; Ardern et al., 2022) with rigorous adherence and was recorded in the PROSPERO registry (CRD42025639229).

### 2.1 Search strategy

Six databases, including PubMed (n = 43), Embase (n = 73), Cochrane Library (n = 106), Web of Science (n = 59), CNKI (n = 80), and EBSCO (n = 129), were systematically searched using computer-based methods to identify randomized controlled trials (RCTs) examining the effects of HIIT and MICT on fat/weight loss and blood pressure in overweight/obese adolescents, from database inception to February 20, 2025. The bibliographies of the selected studies were additionally reviewed manually to uncover any other pertinent literature. The detailed search strategies are outlined as follows: (“HIIT” OR “High-Intensity Interval Training” OR “Interval Training” OR “Intermittent Exercise” OR “Interval Exercise” OR “Intermittent Training”) AND (“MICT” OR “Moderate-Intensity Continuous Training” OR “Endurance Training” OR “Aerobic Training” OR “Moderate-Intensity Exercise” OR “Moderate-Intensity Training”) AND (“Adolescent” OR “Teen” OR “Adolescence” OR “Teenager” OR “Teenagers” OR “Youth” OR “Youths” OR “Young People” OR “Pediatric” OR “Children”) AND (“Overweight” OR “Obese” OR “Obesity” OR “Fat Mass” OR “Weight” OR “BMI” OR “Body Composition” OR “Blood Pressure” OR “Hypertension” OR “Systolic Pressure” OR “Diastolic Pressure” OR “Cardiovascular Health” OR “SBP” OR “DBP” OR “MBP”). Furthermore, the citations of each included study were reviewed to minimize the possibility of relevant articles being missed. Detailed search strategies are presented in Supplementary Tables S1–S6.

### 2.2 Eligibility criteria

Based on the PICO(S) framework (Da Costa Santos et al., 2007), the following inclusion criteria were established:

1. Language: Studies disseminated in English or Chinese.
2. Study type: Randomized controlled trials.
3. Participants: Adolescents aged 10–19 years who were clinically identified as overweight or obese according to WHO standards (BMI-for-age > +1 SD) (Asia WHOROfS-E, 2021; World Health Organization, 2024b), regardless of gender. Individuals with acute or chronic diseases were excluded.
4. Interventions: Studies comparing HIIT and MICT with an intervention duration of at least 3 weeks were included (Johansson et al., 2014; Guerrini Usubini et al., 2022). Moderate intensity was defined as 50%–80% VO_2_max, 60%–75% HRmax, 40%–75% HRR, or 50%–80% MAS, and generally <80% HRpeak. High intensity exercise was described as all-out effort or ≥90% VO_2_peak, ≥80% VO_2_max, 85%–95% HRmax, or ≥100% MAS (Norton et al., 2010; Gibala et al., 2012).
5. Outcome Measures: Key outcomes included body weight, BMI, WC, BF%, and FFM for body composition, as well as SBP and DBP. These indicators are widely recognized in expert consensus for evaluating exercise interventions in adolescent obesity. The American Academy of Pediatrics (AAP) has identified BMI and WC as core indicators of adolescent weight status and metabolic risk, and recommends monitoring obesity-related changes in blood pressure (SE and Expert Committee, 2007). The International Association for the Study of Obesity emphasizes the importance of BF% and WC in identifying central obesity and notes that elevated blood pressure may indicate early-stage obesity-related metabolic syndrome (Lobstein et al., 2015). Additionally, Garber et al. (Garber et al., 2011) highlighted in their exercise intervention guidelines that multi-dimensional indicators, including body fat, FFM, and blood pressure, are essential for comprehensively evaluating the influence of exercise on body composition and cardiovascular fitness. Therefore, these seven parameters were selected as the primary outcome measures in this meta-analysis.

### 2.3 Exclusion criteria

Studies were excluded if they:

1. Included participants with metabolic or chronic disorders affecting weight or cardiovascular function (e.g., diabetes, thyroid dysfunction, sarcopenic obesity).
2. Did not report key outcome measures related to body weight, body composition, or blood pressure (e.g., BMI, body fat, FFM, waist circumference, SBP, DBP).
3. Did not compare HIIT with MICT or had an intervention duration shorter than 3 weeks.
4. Were non-randomized studies.

### 2.4 Screening process and data collection

In accordance with the PRISMA Statement Guidelines for study inclusion and data collection (Moher et al., 2009), the collected literature was administered with EndNote 21 software (Clarivate, Pennsylvania, United States). The software was employed to remove duplicate records. Two researchers (XL and ZC) separately reviewed the literature and gathered data based on the predefined criteria for inclusion and exclusion, followed by cross-validation. If a disagreement arises, a third researcher (ZT) was referred to in order to resolve the issue. If the data is incomplete, the corresponding author was engaged to provide more details. Data extraction was conducted according to predefined criteria, which included: (World Health Organization, 2024a): basic study information such as title, year of publication, author names, and journal; (World Health Organization, 2021); The essential characteristics of the participants, for instance, sample size, gender, age, intervention details, duration, and frequency of exercise in each group; (World Health Organization, 2022); critical bias risk assessment elements.

### 2.5 Literature quality evaluation

Two researchers (XL and ZC) evaluated the quality of the selected studies using the RCT-specific risk of bias tool, as outlined in the Cochrane Handbook (Cumpston et al., 2019). In the event of disagreement and unsuccessful discussion, a third researcher (JL) was consulted for final advice to uphold the reliability and validity of the experimental outcomes. The quality assessment criteria for the included studies as outlined below: (a) Random sequence generation; (b) Allocation concealment; (c) Blinding of participants and personnel; (d) Blinding of outcome assessment; (e) Incomplete outcome data; (f) Selective reporting; (g) Other bias. Each domain was rated as low risk, high risk, or unclear risk (Savović et al., 2012).

### 2.6 Data synthesis and analysis

Excel (Office v.2021, Microsoft Corp) was used for data summarization, Review Manager 5.4 (Cochrane Collaboration, Oxford, United Kingdom) for data processing and analysis, and Stata MP version 18 (Stata Corp) for generating funnel plots. In all studies, HIIT was designated as the experimental group, with MICT serving as the comparison group. Forest plots were generated using post-intervention means and standard deviations. The mean difference was calculated as HIIT minus MICT, irrespective of the display order of the groups in the plot. When the measurement methods and units of the outcome indices are the same, the mean difference (MD) is utilized as the effect size; if there is a difference, the standardized mean difference (SMD) is applied, with a 95% confidence interval (95% CI) used as the statistical range. Regarding heterogeneity, when I^2^ < 50%, medium or low heterogeneity is indicated, and the fixed-effect model was applied for meta-analysis (Page et al., 2021). When I^2^ ≥ 50%, significant heterogeneity was observed, and the random-effects model was applied for meta-analysis (Hosseini et al., 2012), with a significance threshold set at α = 0.05 (Higgins et al., 2003; Higgins, 2008). To determine the causes of heterogeneity, the selected studies were sorted by exercise intervention type (running, cycling, or other), load-rest ratio (<1 and ≥1), and intervention duration (<12 weeks and ≥12 weeks) in the subgroup analysis. The grouping followed classification criteria from previous systematic reviews and meta-analyses (Weston and Coombes, 2014; Cao et al., 2021; Costigan et al., 2015), aiming to strengthen the explanatory power and practical usability of the findings. Sensitivity analysis (using the one-by-one elimination method) was performed to assess the reliability of the outcomes. Finally, the eligible indicators were used to generate a funnel plot for assessing publication bias. In addition, exploratory subgroup analyses were conducted *post hoc*, based on intervention characteristics, to further investigate potential sources of heterogeneity.

## 3 Results

### 3.1 Process of selecting studies

**FIGURE 1:**
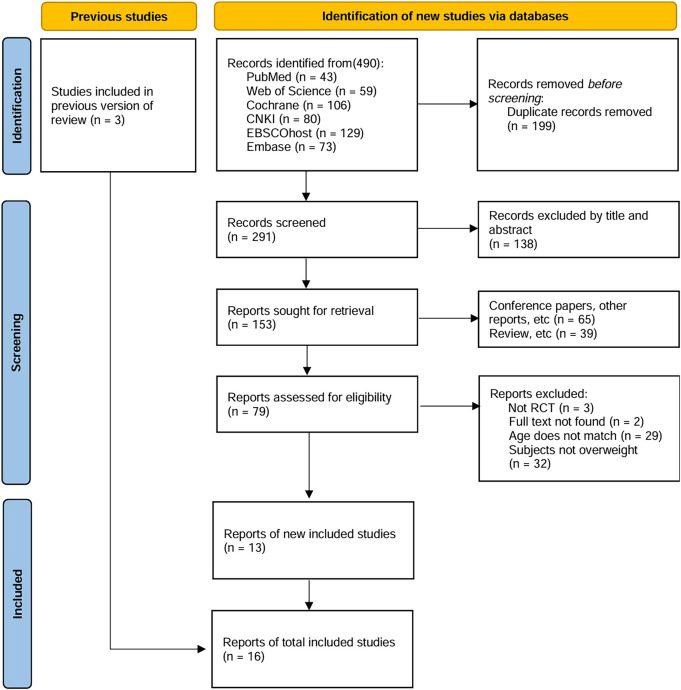
Study selection flowchart in PRISMA.

Figure 1 depicts the literature inclusion process, culminating in 490 eligible studies after applying rigorous exclusion filters from six databases using the search formula. 199 duplicate articles were excluded using EndNote 21 software, and 138 studies were further excluded on the grounds of title and abstract. Of the remaining 153 articles, 65 were conference papers or other types of publications, 39 were review articles, 3 were not randomized controlled trials, 2 had missing full texts, 29 were not age-matched, and 32 involved non-obese/overweight individuals. Additionally, by tracing the references of previous reviews, three studies meeting the criteria were included. Ultimately, 16 studies were included, with multiple outcome indicators being assessed (Cao et al., 2022; Li et al., 2023; Corte de Araujo et al., 2012; Cvetkovic et al., 2018; Koubaa et al., 2013; Leite et al., 2022; Meng et al., 2022; Morrissey et al., 2018; Wen and Lou, 2018; Bahreini et al., 2021; Dias et al., 2018; Fernandez et al., 2004; Julian et al., 2022; Lazzer et al., 2017; Miguet et al., 2020).

### 3.2 Study parameters

Table 1 systematically compiles the fundamental parameters and experimental outcomes derived from individual investigations in this quantitative review. A total of 16 RCTs, released from 2004 to 2023, originated from 10 different countries: Iran, China, Brazil, Spain, Australia, France, Tunisia, Italy, and the United States. Participant cohorts varied between 16 and 65 subjects per experimental group, culminating in an aggregate enrollment of 473 research-eligible individuals. The interventions included running, power cycling, and combined exercise. Among them, 2 exercise intervention cycles lasted 16 weeks (Julian et al., 2022; Miguet et al., 2020), 11 lasted 12 weeks (Cao et al., 2022; Li et al., 2023; Corte de Araujo et al., 2012; Cvetkovic et al., 2018; Koubaa et al., 2013; Leite et al., 2022; Meng et al., 2022; Morrissey et al., 2018; Wen and Lou, 2018; Dias et al., 2018; Fernandez et al., 2004; Starkoff et al., 2014), and the remaining 3 RCTs had intervention cycles of 8 weeks, 6 weeks, and 3 weeks, respectively (Bahreini et al., 2021; Julian et al., 2022; Starkoff et al., 2014). Only three trials had a frequency of 2 sessions per week (Corte de Araujo et al., 2012; Julian et al., 2022; Miguet et al., 2020), while the others had 3 sessions per week. More than half of the work-to-rest ratios were ≥1 (Cao et al., 2022; Li et al., 2023; Cvetkovic et al., 2018; Koubaa et al., 2013; Meng et al., 2022; Morrissey et al., 2018; Dias et al., 2018; Julian et al., 2022; Miguet et al., 2020; Starkoff et al., 2014). All 16 trials used body weight and BMI as outcome measures. Additionally, each of the 16 trials incorporated body weight, BMI, and BF% in their evaluations. 8 trials used waist circumference as an outcome measure (Cao et al., 2022; Li et al., 2023; Corte de Araujo et al., 2012; Koubaa et al., 2013; Leite et al., 2022; Meng et al., 2022; Wen and Lou, 2018; Starkoff et al., 2014), 12 trials used fat-free mass (Cao et al., 2022; Li et al., 2023; Corte de Araujo et al., 2012; Cvetkovic et al., 2018; Koubaa et al., 2013; Leite et al., 2022; Meng et al., 2022; Morrissey et al., 2018; Dias et al., 2018; Julian et al., 2022; Lazzer et al., 2017; Miguet et al., 2020), and 8 studies included systolic and diastolic blood pressure as key indicators (Cao et al., 2022; Li et al., 2023; Corte de Araujo et al., 2012; Cvetkovic et al., 2018; Koubaa et al., 2013; Leite et al., 2022; Meng et al., 2022; Morrissey et al., 2018; Dias et al., 2018).

**TABLE 1:** Study features of those included in the meta-analysis.

Study	Year	Country	Total(H/M)	Age(M±SD)	Modality	Exercise Intensity (HIIT) (MICT)	Load-rest ratio	Frequency	Exercise period	outcome measure
Bahreini et al. (2021)	2021	Iran	23(12/11)	9–11	NR	(70%-80%VO_2_max) (70%–80% VO_2_max)	NR	3 days/week	8 weeks	①②⑤
Cao et al. (2022)	2022	China	33(17/16)	10.8 ± 0.7	Running	(90%-100%MAS) (60%–70% MAS)	15s/15s (1.00)	3 days/week	12 weeks	①②③④⑤⑥⑦
Corte de Araujo et al. (2012)	2012	Brazil	30(15/15)	8–12	Running	(100%MAS) (80% HRpeak)	60s/180s (0.33)	2 days/week	12 weeks	①②③④⑤⑥⑦
Cvetkovic et al. (2018)	2018	Serbia	21(11/10)	11–13	Running	(100%MAS) (75% HRmax)	10–20s/10–20s (1.00)	3 days/week	12 weeks	①②④⑤⑥⑦
Davis et al. (2006)	2018	Australia	41(17/24)	7–16	Running	(85–95%HRmax) (60%–70% HRmax)	240s/180s (1.33)	3 days/week	12 weeks	①②④⑤
Fernandez et al. (2004)	2004	Brazil	19(10/9)	15–19	Cycling	(NR) (NR)	30s/180s (0.17)	3 days/week	12 weeks	①②⑤
Julian et al. (2022)	2021	French	38(19/19)	12–16	Cycling	(75%-90%VO_2_peak) (60% VO_2_peak)	30s/30s (1.00)	2 days/week	16 weeks	①②④⑤
Koubaa et al. (2013)	2013	Tunis	29(14/15)	13.0 ± 0.8	Running	(80%-90%vVO_2_max) (60%-70%vVO_2_max)	120s/60s (2.00)	3 days/week	12 weeks	①②③④⑤⑥⑦
Lazzer et al. (2017)	2016	Italy	30(10/20)	15–17	Running	(100%VO_2_max) (70% VO_2_max)	40s/300s (0.13)	3 days/week	3 weeks	①②④⑤
Leite et al. (2022)	2022	Brazil	40(20/20)	10–15	Running and Cycling	(100%MAS) (35%–75% HRR)	30s/45s (0.67)	3 days/week	12 weeks	①②③④⑤⑥⑦
Li et al. (2023)	2023	China	31(16/15)	11.0 ± 0.8	Running	(100%-120%MAS) (60%–80% MAS)	15s/15s (1.00)	3 days/week	12 weeks	①②③④⑤⑥⑦
Meng et al. (2022)	2022	China	23(12/11)	11.2 ± 0.7	Running	(90%-100%MAS) (60%–70% MAS)	15s/15s (1.00)	3 days/week	12 weeks	①②③④⑤⑥⑦
Miguet et al. (2020)	2019	French	43(22/21)	13.6 ± 1.5	Cycling	(75%-90%VO_2_peak) (60% VO_2_peak)	30s/30s (1.00)	2 days/week	16 weeks	①②④⑤
Morrissey et al. (2018)	2018	French	29(16/13)	12–16	Combined motion	(90%-95%HRmax) (60%–70% HRmax)	120s–150s/90s (1.33)	3 days/week	12 weeks	①②④⑤⑥⑦
Starkoff et al. (2014)	2014	America	27(14/13)	14.7 ± 1.5	Cycling	(90%-95%APMHR) (65%–70% APMHR)	120s/60s (2.00)	3 days/week	6 weeks	①②③⑤
Wen and Lou (2018)	2018	China	16(8/8)	10–12	Combined motion	(85%-95%HRmax) (60%–70% HRmax)	90s/180s (0.33)	3 days/week	12 weeks	1 ②③⑤

HIIT: High-intensity interval training; MICT: Moderate-intensity continuous training; H: HIIT, group; M: MICT, group; VO_2_max: Maximal Oxygen Uptake; MAS: maximal aerobic speed; HRpeak: Peak Heart Rate; NR: not reported; HRmax: Maximum Heart Rate; VO_2_peak: Peak Oxygen Uptake; vVO_2_max: Velocity at Maximal Oxygen Uptake; HRR: heart rate reserve; APMHR: Age-Predicted Maximum Heart Rate; ①Body mass; ②Body mass index; ③Waist circumference; ④Fat-free mass; ⑤Body fat percentage; ⑥Systolic blood pressure; ⑦Diastolic blood pressures.

### 3.3 Risk of bias

**FIGURE 2:**
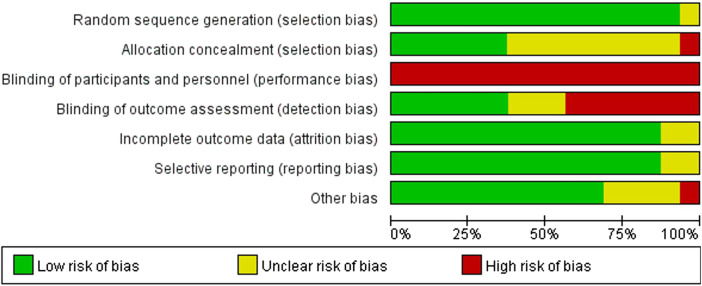
Risk of bias graph.

Consistent with the PRISMA guidelines, a formal risk of bias assessment was conducted for all included studies. Figure 2 presents the risk of bias evaluation, which was conducted by applying the Cochrane risk of bias assessment tool to appraise the methodological reliability of the included trials. Regarding the generation of random sequences to address selection bias, one study was unclear (Corte de Araujo et al., 2012), while the remaining 15 studies were assessed as low risk (Cao et al., 2022; Li et al., 2023; Cvetkovic et al., 2018; Koubaa et al., 2013; Leite et al., 2022; Meng et al., 2022; Morrissey et al., 2018; Wen and Lou, 2018; Bahreini et al., 2021; Dias et al., 2018; Fernandez et al., 2004; Julian et al., 2022; Lazzer et al., 2017; Miguet et al., 2020). For allocation concealment (selection bias), 1 study was rated as high risk (Morrissey et al., 2018), 9 studies as unclear (Cao et al., 2022; Corte de Araujo et al., 2012; Cvetkovic et al., 2018; Koubaa et al., 2013; Leite et al., 2022; Wen and Lou, 2018; Bahreini et al., 2021; Fernandez et al., 2004; Lazzer et al., 2017), and 6 studies as low risk (Li et al., 2023; Meng et al., 2022; Dias et al., 2018; Julian et al., 2022; Miguet et al., 2020). Concerning the masking of participants and study investigators to minimize intervention bias, owing to the practical limitations associated with exercise interventions, blinding of all adolescents’ post-randomization is challenging. Furthermore, researchers may be unable to blind the interventions, potentially affecting both intervention implementation and the interpretation of results; thus, each of the 16 studies was found to be at high risk of bias. Concerning the use of blinding to assess outcomes (detection bias), 7 studies were considered high risk (Li et al., 2023; Leite et al., 2022; Meng et al., 2022; Morrissey et al., 2018; Wen and Lou, 2018; Miguet et al., 2020), 3 studies were unclear (Cvetkovic et al., 2018; Bahreini et al., 2021), and 6 studies were low risk (Corte de Araujo et al., 2012; Koubaa et al., 2013; Dias et al., 2018; Fernandez et al., 2004; Julian et al., 2022; Lazzer et al., 2017). In evaluating attrition bias and reporting bias, associated with missing data and selective disclosure, respectively, 14 studies were low risk (Cao et al., 2022; Li et al., 2023; Cvetkovic et al., 2018; Koubaa et al., 2013; Leite et al., 2022; Meng et al., 2022; Morrissey et al., 2018; Wen and Lou, 2018; Bahreini et al., 2021; Dias et al., 2018; Julian et al., 2022; Lazzer et al., 2017; Miguet et al., 2020), while the remaining 2 studies were unclear (Corte de Araujo et al., 2012; Fernandez et al., 2004). Finally, for other biases, 1 study was rated as high risk (Fernandez et al., 2004), 4 studies were unclear (Corte de Araujo et al., 2012; Morrissey et al., 2018; Wen and Lou, 2018; Bahreini et al., 2021), and 11 studies were low risk (Cao et al., 2022; Li et al., 2023; Cvetkovic et al., 2018; Koubaa et al., 2013; Leite et al., 2022; Meng et al., 2022; Dias et al., 2018; Julian et al., 2022; Lazzer et al., 2017; Miguet et al., 2020; Higgins et al., 2024).

### 3.4 Publication bias

**FIGURE 3:**
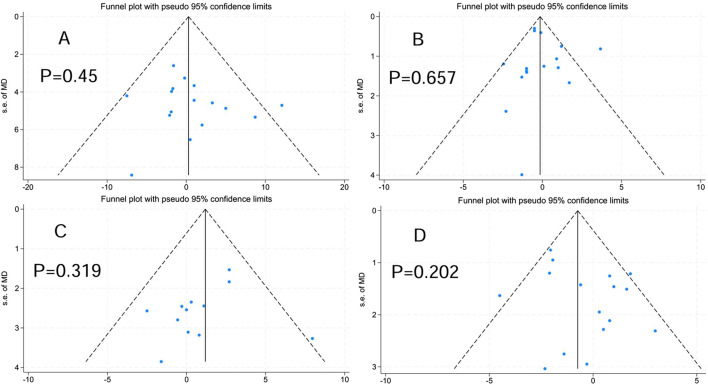
Publication bias. **(A)** Body mass. **(B)** Body mass index. **(C)** Fat-free mass. **(D)** Body fat percentage.

As stated by the recommendations of the Cochrane Handbook, funnel plots and Egger’s test were performed on indicators with data from 10 or more studies to evaluate publication bias, and bias was assumed when the p-value met or fell below the 0.05 threshold (Higgins et al., 2024). As shown in Figure 3, Body mass (bias = 0.77, P = 0.45 > 0.05), BMI (bias = 0.305, P = 0.657 > 0.05), FFM (bias = −1.127, P = 0.319 > 0.05), BF% (bias = 1.113, P = 0.202 > 0.05).

### 3.5 Body composition

#### 3.5.1 Body weight

**FIGURE 4:**
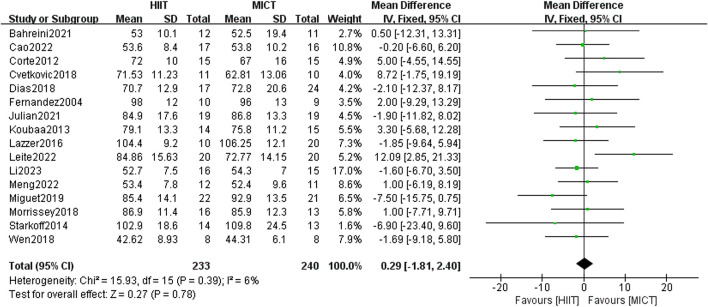
Body mass of forest plot.

The 16 included randomized controlled trials compared the difference in body weight (n = 16) between the HIIT group and the MICT group. The findings from the fixed-effect model meta-analysis revealed no statistical significance [MD = 0.29, 95% CI (−1.81, 2.40), Z = 0.27, P = 0.78] (Figure 4). After conducting a sensitivity analysis, the differences remained non-significant after excluding studies one by one. Due to its low heterogeneity (I^2^ = 6%, p > 0.05), the results showed strong consistency, though the overall effect size did not reach statistical significance.

#### 3.5.2 Body mass index (BMI)

**FIGURE 5:**
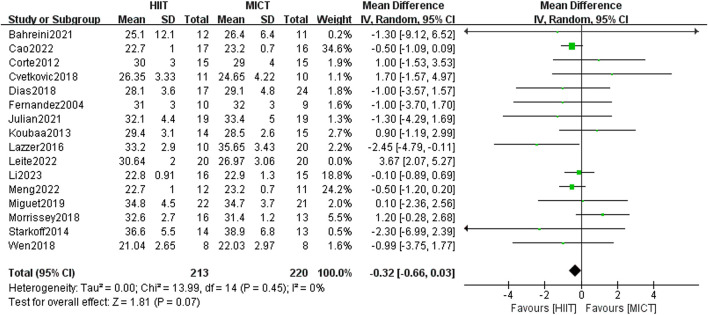
Body mass index of forest plot.

Sixteen RCTs compared the influence of HIIT and MICT on BMI. Results from the random-effects model meta-analysis showed no significant difference between the two groups [MD = 0.06, 95% CI (−0.62, 0.74), Z = 0.17, P = 0.87], with large heterogeneity (I^2^ = 59%, p > 0.05). After conducting sensitivity analysis, studies were excluded one by one, and it was found that when Leite et al. (2022) (published in 2022) was excluded, the heterogeneity decreased [MD = −0.32, 95% CI (−0.66, 0.03), Z = 1.81, P = 0.07], with I^2^ = 0%, p > 0.05. However, the intervention effects between the two groups exhibited no significant difference (Figure 5).

#### 3.5.3 Waist circumference (WC)

**FIGURE 6:**
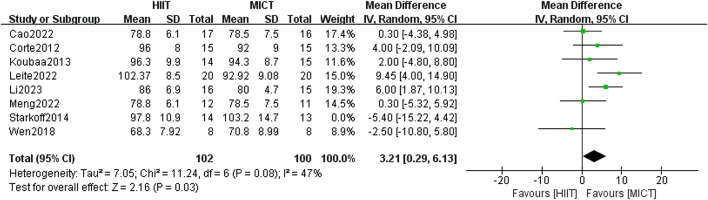
Waist circumference of forest plot.

16 studies investigated the influence of HIIT and MICT on WC. There was no meaningful difference between the two groups found in the random-effects model meta-analysis [MD = 2.58, 95% CI (−0.43, 5.59), Z = 1.68, P = 0.09]. However, after removing the study by Starkoff et al. (Starkoff et al., 2014) (published in 2014), which had high heterogeneity, the difference between the two groups was found to be statistically significant [MD = 3.21, 95% CI (0.29, 6.13), Z = 2.16, P = 0.03] (Figure 6), with the heterogeneity reduced to less than 50% (I^2^ = 47%, p < 0.05). Given the high overall heterogeneity (I^2^ = 51%, p > 0.05), a subgroup analysis was undertaken to examine potential factors contributing to heterogeneity. The results indicated that when running was used as the form of exercise, MICT showed greater effectiveness than HIIT in improving WC in overweight/obese adolescents [MD = 2.81, 95% CI (0.36, 5.26), Z = 2.25, P = 0.02]. However, for other forms of exercise (such as power cycling and combined training), variation in WC improvement was found between the two groups. The heterogeneity was high (I^2^ = 79%), although it was not statistically significant (p = 0.82) (Table 2). In the sensitivity analysis, removal of the study by Leite et al. (Leite et al., 2022) showed that the heterogeneity decreased markedly (I^2^ = 0%, p = 0.25). Notably, Leite et al. used the waist-to-height ratio (WtHR) to adjust for height, while Starkoff et al. employed a standard anthropometric ruler to measure the midpoint and maximum protrusion between the lowest rib and the hip bone. These differing measurement methods could account for methodological variations in the results. Furthermore, Starkoff et al. adopted the highest exercise intervention load-rest ratio of 2.00 among the 16 RCTs, which may have contributed to the large discrepancy between their results and those of other studies.

**TABLE 2:** Waist circumference (WC) of subgroup analyses results.

Included studies	WMD	95%CI	I^2^	p
Running				
4	−0.51	[-4.08,3.07]	0	0.78
4	4.87	[1.11,8.63]	0.55	0.01
Others				
5	2.81	[0.36,5.26]	8%	0.02
3	1.12	[-8.72,10.97]	79%	0.82

#### 3.5.4 Fat-free mass (FFM)

**FIGURE 7:**
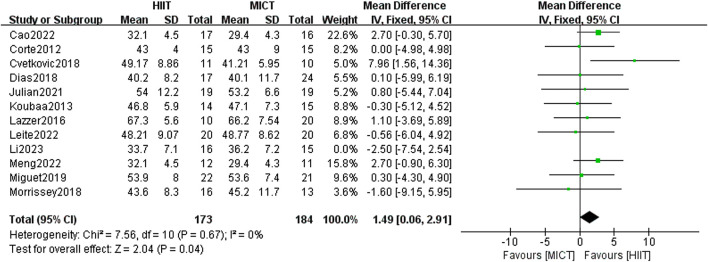
FFM of forest plot.

In total, 12 studies were included to examine the influence of HIIT and MICT on FFM. Using the fixed-effect model, the meta-analysis found no meaningful difference between the two groups [MD = 1.19, 95% CI (−0.19, 2.56), Z = 1.70, P = 0.09]. Given the absence of heterogeneity (I^2^ = 0%, p > 0.05), the results were consistent. After conducting a sensitivity analysis, it was found that the results became statistically significant when the study by Li et al. (Li et al., 2023) (published in 2023) was excluded [MD = 1.49, 95% CI (−0.06, 2.91), Z = 2.04, P = 0.04] (Figure 7), with no heterogeneity remaining at this point (I^2^ = 0%, p < 0.05). Most of the randomized controlled trials included in the analysis employed the dual-energy X-ray absorption technique (DXA, Lunar Prodigy, United States) in standard mode to measure FFM. A few studies employed bioelectrical impedance (Biodynamics®) to measure body composition and calculate FFM. Since Li et al. used the DXA method, measurement differences could be a potential explanation for the observed disparity, though further investigation is limited due to the lack of additional supporting evidence.

#### 3.5.5 Body fat percentage (BF%)

**FIGURE 8:**
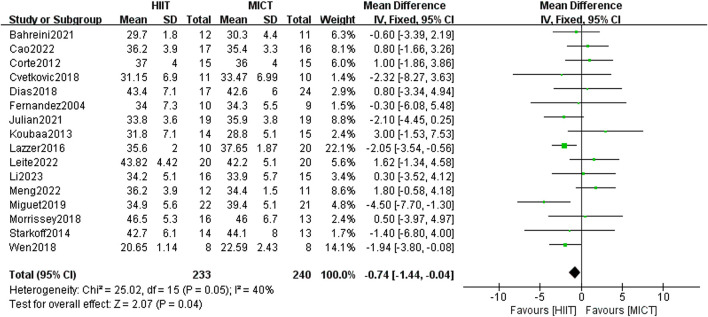
Body fat percentage of forest plot.

The analysis encompassed 16 studies, comparing HIIT and MICT in terms of their effects on BF%. The meta-analysis using the fixed-effect model showed a statistically significant difference between the experimental group and the comparison group [MD = −0.74, 95% CI (−1.44, 0.04), Z = 2.74, P = 0.04] (Figure 8), with low heterogeneity (I^2^ = 40%, p < 0.05). Therefore, HIIT demonstrated greater efficacy than MICT in reducing the BF% of overweight/obese adolescents.

### 3.6 Blood pressure (BP)

#### 3.6.1 SBP

**FIGURE 9:**
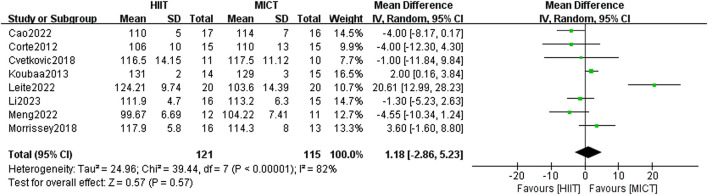
SBP of forest plot.

A total of 8 RCTs assessed the impact of HIIT and MICT on SBP. The results of the random-effects model yielded results indicating no appreciable difference between the two groups [MD = 1.18, 95% CI (−2.86, 5.23), Z = 0.57, P = 0.57] (Figure 9). However, the heterogeneity was extremely high (I^2^ = 82%, p > 0.05), prompting a subgroup analysis to explore potential factors of heterogeneity. Despite this, heterogeneity within subgroups remained high. Examination of the forest plot indicated that the study by Leite et al. (2022) showed a markedly larger effect size (mean difference = 20.61 mmHg, 95% CI: 12.99–28.23), substantially influencing both the pooled estimate and overall heterogeneity. Notably, in Leite et al.’s study, the HIIT group engaged in a significantly shorter exercise duration (15–18 min per session), compared to the MICT group’s 90 min per session, resulting in markedly unbalanced energy expenditure, which may explain its status as a statistical outlier.

Furthermore, variability in blood pressure measurement methods across studies may have contributed to the observed heterogeneity. For example, Meng et al. (2022). used an automatic BP monitor (Omron BP652, United States) after 10 h of fasting and a 5-min rest period, while Leite et al. (2022) measured BP after fasting for 12 h and resting for 10 min using a manual sphygmomanometer, with cuff sizes adjusted to participants’ arm circumference. Differences in equipment, the number of measurements taken, and environmental conditions likely influenced the reported outcomes. The lack of standardized blood pressure measurement protocols across studies is thus a critical element contributing to the substantial heterogeneity in study results (I^2^ = 82%).

#### 3.6.2 DBP

**FIGURE 10:**
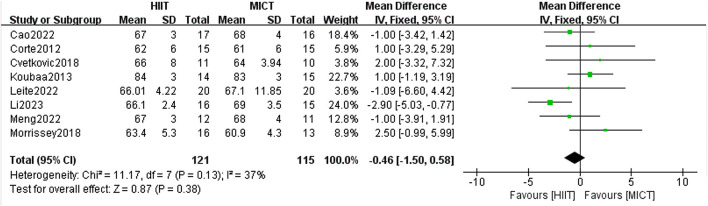
DBP of forest plot.

8 RCTs analyzed the impact of HIIT and MICT on DBP, and the fixed-effect model meta-analysis showed no significant difference between the two groups [MD = −0.46, 95% CI (−1.50, 0.58), Z = 0.87, P = 0.38] (Figure 10). The robustness of the result was confirmed through a sensitivity analysis by sequentially excluding individual studies, as no significant changes were observed. The heterogeneity among studies was low (I^2^ = 37%, p > 0.05), indicating consistent findings across the included trials. Notably, although the difference was not statistically significant, a general trend favoring HIIT over MICT in reducing DBP was observed in most studies. This non-significant trend suggests that HIIT may have a slightly greater potential for lowering DBP in overweight or obese adolescents, warranting further investigation in future trials with larger sample sizes or longer intervention durations.

## 4 Discussion

In our review and pooled analysis, 16 RCTs with 473 overweight or obese adolescents were included to assess the influence of HIIT and MICT on body weight, composition of body mass, and blood pressure. HIIT demonstrated a significantly higher effectiveness than MICT in reducing body fat percentage, although both interventions showed no significant differences in body weight, BMI, FFM, SBP, or DBP. The subgroup analysis indicated that MICT was more effective in reducing WC, particularly when running was used as the primary form of intervention. Despite the overall findings, sensitivity analyses demonstrated that the results were influenced by individual studies, possibly attributable to the small number of trials included. These results emphasize the potential advantages of HIIT in improving body composition, while also stressing the necessity for further large-scale, high-quality RCTs to bolster the evidence and increase the reliability of the findings.

### 4.1 Effects of HIIT and MICT on body mass and composition in obese adolescents

With the progression of modern society and changes in lifestyle, the rate of overweight and obesity has risen considerably. These conditions are associated with a range of comorbidities, prompting extensive research into effective strategies for fat reduction and health improvement. MICT has long been recognized as a traditional and effective method for enhancing physical activity and health outcomes (Miles, 2007). In recent years, HIIT has gained growing attention for its capacity to swiftly deplete glycogen stores and enhance fat oxidation and lipolysis (Peake et al., 2014), making it an emerging strategy for fat loss. It is well-established that both HIIT and MICT positively impact the composition of body mass in overweight/obese adolescents, including reductions in BF%, BMI, and WC, as well as increases in FFM (Atakan et al., 2021).

Our meta-analysis demonstrated that HIIT and MICT bring about comparable effects on body weight and general body composition. Notably, HIIT showed a greater effect in reducing BF%, which aligns with the findings of Poon et al. (Poon et al., 2024), who reported that this advantage is particularly evident in protocols with longer intervention durations, cycling-based training, and lower HIIT volume. Subgroup analysis in our study revealed that MICT was more effective than HIIT in reducing WC, particularly when running was the primary mode of exercise. Sensitivity analysis further indicated that HIIT had a statistically significant advantage over MICT in improving FFM after excluding a study with high heterogeneity. These results align with Wewege et al. (2017), who also reported that WC reductions induced by MICT may range from 1 to 7 cm, especially when running is involved. The change in FFM appears to be closely linked to BF% reduction, as HIIT typically promotes fat loss while simultaneously stimulating muscle hypertrophy. This dual effect is more pronounced in high-intensity protocols, contributing not only to improved body composition but also to enhanced physical health and appearance (Osawa et al., 2014). Thus, the increase in FFM may be a key contributor to the observed reduction in BF%. Despite these findings, inconsistencies remain in the literature. Some previous reviews have concluded that two types of exercise have comparable impacts on body tissue composition—specifically BF%, WC, and FFM—with the primary difference being time efficiency. HIIT has been shown to achieve comparable fat loss outcomes in a significantly shorter duration than MICT (Cao et al., 2021; Wewege et al., 2017; Osawa et al., 2014; Sultana et al., 2019).

We believe that the discrepancies in findings among various studies may be attributed to several key factors. First, most existing trials focus on overweight or obese adults, including college students, middle-aged adults, and the elderly. In contrast, there is a notable shortage of comparative studies focused on adolescents. However, physiological responses to exercise can vary significantly by age group. Adolescents, being in a period of rapid growth and development, exhibit heightened secretion of somatotropin, insulin-like growth factor, and testosterone—hormones that promote both lipolysis (especially of visceral fat) and muscle synthesis (Christoforidis et al., 2005). Consequently, adolescents often experience more pronounced fat reduction following exercise interventions. In contrast, adults typically show age-related declines in hormone levels, reduced fat oxidation capacity, and diminished visceral fat sensitivity to exercise stimuli, resulting in more modest reductions in adiposity (Mancuso and Bouchard, 2019; Frandsen et al., 2021). Second, some studies included both overweight/obese and normal-weight individuals or were conducted exclusively in healthy populations. This lack of specificity reduces the applicability of results to the overweight/obese subgroup. In contrast to individuals with normal weight, overweight/obese individuals store more adipose tissue, suffer from greater insulin resistance, and have impaired mitochondrial function, leading them to utilize carbohydrates more heavily for energy during exercise (Kwak, 2013; Braun et al., 1985; Højlund et al., 2008). HIIT can notably boost fat oxidation following exercise during the period of excess post-exercise oxygen consumption (EPOC) phase by stimulating catecholamine release, thereby promoting more efficient lipolysis (Jiang et al., 2024; Islam et al., 2018). Conversely, during MICT, normal-weight individuals tend to rely more on fat for energy, whereas in HIIT, sympathetic nervous system activation and rapid lactic acid accumulation inhibit fatty acid transport and oxidation, shifting energy reliance back to carbohydrates (Aslankeser and Balcı, 2017; Bruce et al., 2006; Hetlelid et al., 2015). Moreover, because normal-weight individuals generally have better mitochondrial function and smaller energy deficits, the scope for improving fat oxidation efficiency is limited. Additionally, muscle anabolism induced by HIIT in this population may offset fat loss, leading to minimal changes in body fat percentage (Davis et al., 2006).

Existing meta-analyses have demonstrated that 12 weeks of HIIT can reduce visceral fat by 17% and systolic blood pressure by 8 mmHg in obese individuals (Sijie et al., 2012), while in normal-weight populations, HIIT typically reduces body fat percentage by 2%–4% with no significant impact on body weight (Boutcher, 2011). These differences underscore the impact of metabolic status, hormonal environment, inflammation, and cardiovascular function on exercise outcomes, with overweight/obese individuals deriving more pronounced benefits from HIIT. Furthermore, methodological differences in HIIT implementation may contribute to inconsistent findings. Some studies adopted low-volume HIIT protocols (i.e., total exercise ≤500 metabolic equivalent of task-minutes/week) or sprint interval training (SIT) as the intervention, comparing them with MICT. In theory, standard HIIT protocols expend more calories than low-volume HIIT under equivalent conditions. A meta-analysis by Sultana et al. (Sultana et al., 2019) involving 47 studies and 1,422 participants found that low-volume HIIT did not show a significant increase in adiposity, fat mass, or lean body mass when compared to MICT, highlighting the variability in exercise dose and its implications. Lastly, individual dietary habits are a critical but often uncontrolled factor influencing study outcomes (Hooper et al., 2012; Chawla et al., 2020). Certain studies suggest that HIIT may curb caloric intake by modulating appetite-related hormonal pathways such as leptin, whereas no such effect has been observed in MICT or control groups (Sim et al., 2015). This appetite-suppressing effect may further explain why HIIT can lead to more significant reductions in BF% compared to MICT.

It is worth noting that most assessments of body composition in the studies included were conducted employing methods like bioelectrical impedance analysis (BIA), dual-energy X-ray absorptiometry (DEXA), and magnetic resonance imaging (MRI). While these methods are widely accepted and commonly used, each carries inherent limitations and potential measurement errors (Ward, 2019), which may have helped lead to variability in research findings and affected the consistency of the results.

### 4.2 Effects of HIIT and MICT on blood pressure in obese adolescents

Hypertension is a significant contributor to the development of cardiovascular disease (CVD) (Fryar CDC and Li, 2012), predominantly affecting older populations. However, elevated blood pressure in youth may serve as a precursor to essential hypertension later in life (Ortega et al., 2008). Obesity-related reductions in cardiorespiratory fitness and excessive visceral fat accumulation are also independent contributors to CVD risk (Neeland et al., 2019; Henriksson et al., 2019). Therefore, early intervention to lower blood pressure is especially important for adolescents, particularly in individuals who are overweight/obese. Previous research has demonstrated that both HIIT and MICT can contribute to enhanced cardiovascular health among adolescents (Su et al., 2024; Ingul et al., 2018), and it has been reported that a reduction of SBP by more than 4 mmHg could potentially lower cardiovascular mortality by 5%–20% (Taylor et al., 2011). Additionally, WC shows a positive correlation with SBP in children with obesity (Zampetti et al., 2018). In our meta-analysis, there was no statistically significant difference in the effects of HIIT and MICT on SBP and DBP among overweight/obese adolescents. Nevertheless, five of the eight included randomized controlled trials reported that HIIT demonstrated greater efficacy than MICT in lowering SBP. Regarding DBP, the results were more evenly split, with half of the studies favoring HIIT and the other half supporting MICT. These findings indicate ongoing debate and the need for further investigation into the comparative cardiovascular benefits of these two exercise modalities in this population.

For example, Wang et al. (2024) carried out a meta-analysis including 468 adolescents and concluded that HIIT led to a more pronounced reduction in SBP compared to MICT (SMD = −0.35, 95% CI: −0.78 to 0.09). Similarly, García-Hermoso et al. (2016) found that a 4–12-week HIIT intervention in 274 overweight/obese youths led to a greater reduction in SBP compared to other forms of exercise training (SMD = 0.39; equivalent to a 1.33 mmHg decrease). Moreover, several studies have suggested that HIIT may outperform MICT not only in reducing DBP but also in enhancing resting heart rate (HR). For instance, Cheema et al. (Cheema et al., 2015) implemented a 12-week intervention in obese adults and found that HIIT reduced resting HR by an average of 4 beats per minute and DBP by approximately 7 mmHg, whereas MICT reduced HR by only 1 beat per minute with no significant change in DBP. These findings imply that, while the impact of HIIT on DBP appears to be less consistent across studies, it may still offer advantages in cardiovascular regulation. The differential effects of HIIT on SBP and DBP may be partly explained by underlying physiological mechanisms. SBP reflects arterial pressure during cardiac contraction and is primarily influenced by cardiac output and arterial elasticity (Shahoud JSS and Aeddula, 2023). HIIT can improve SBP by enhancing cardiac function, increasing arterial compliance, and reducing vascular resistance (Luo et al., 2024). In contrast, DBP represents the arterial pressure during cardiac relaxation, primarily determined by the resistance of small arteries and peripheral vascular tone, and is more influenced by the regulation of microvascular function (Safar, 2004; Jackson, 2021). These differences may explain why improvements in SBP are more frequently observed and statistically significant in HIIT interventions, while changes in DBP tend to be smaller and more variable.

On the other hand, HIIT primarily influences the function of the aorta and the heart’s pumping capacity, enhancing cardiac contractility, cardiac output, and the elasticity or compliance of major arteries, including the aorta and carotid artery (O'Driscoll et al., 1985). These adaptations are more directly associated with reductions in SBP. In contrast, DBP is more dependent on arteriolar tone, sympathetic nervous system activity, and resting vascular tension—factors that typically require longer or more cumulative physiological stimuli to elicit adaptive changes (Incognito et al., 2019). Cardiac diastolic function is also crucial in regulating blood pressure. Interestingly, multiple studies have indicated that HIIT outperforms MICT in enhancing diastolic function (Gonçalves et al., 2024). Moreover, the duration of exercise intervention is a crucial factor influencing outcomes. Many included trials employed intervention periods ranging from 4 to 12 weeks, which may be insufficient to significantly affect microvascular remodeling or endothelial function—both of which are necessary for meaningful reductions in DBP. Additionally, adolescent-specific characteristics can further complicate DBP regulation. Overweight and obese adolescents may exhibit microvascular abnormalities or insulin resistance that blunt improvements in vascular function. Furthermore, during puberty, hormonal fluctuations and heightened sympathetic activity make DBP more susceptible to external factors such as emotional stress, sleep disturbances, and lifestyle patterns (Urbina et al., 2012; Seravalle et al., 2018). Therefore, SBP tends to show more immediate improvements in response to exercise interventions, while changes in DBP may require longer-term or more targeted strategies.

Some research has found that physical activity of different intensities significantly improved SBP and DBP. For example, Way et al. (2019) reported that HIIT led to a significant reduction in nighttime diastolic blood pressure (effect size: −0.456, 95% CI: −0.826 to −0.086 mmHg; p = 0.016), and also observed a trend toward daytime reductions in both DBP and SBP (ES: −0.349, 95% CI: −0.717 to 0.020 mmHg; p = 0.063). While much of the early evidence was derived from adult populations, more recent research has increasingly focused on children and adolescents. Cornelissen and Smart (2013) found that aerobic endurance training significantly reduced both SBP and DBP by an average of −3.5 mmHg (95% CI: −4.6 to −2.3 mmHg), and subgroup analyses suggested that interventions characterized by running, longer training durations, older participant age, and shorter daily training times (<24 min) were more effective in lowering blood pressure in youth populations. Zhou et al. (2025) performed a meta-analysis that included 19 RCTs with a total of 819 overweight or obese children and adolescents. The analysis found that exercise interventions led to a significant reduction in SBP (SMD = −0.71, 95% CI: −1.06 to −0.36, p < 0.001) and DBP (SMD = −0.67, 95% CI: −1.00 to −0.34, p < 0.001). The effects were particularly pronounced among girls, obese participants, those involved in HIIT programs, and individuals who accumulated more than 3000 min of total exercise time. These results stress the importance of implementing early exercise interventions to prevent or delay hypertension in individuals at high risk. Similarly, Cao et al. (2021) meta-analysis highlighted that interventions lasting longer than 8 weeks and employing running as the primary mode of exercise were particularly effective in reducing blood pressure among children around 10 years old.

The differing conclusions regarding the impact of HIIT and MICT on blood pressure could be explained by differences in baseline blood pressure levels among the study populations. Some studies focus primarily on the “average blood pressure reduction,” while others use the criterion of “whether blood pressure reaches the normal range.” Additionally, measurement discrepancies, such as differences in equipment, timing of measurements, and environmental controls, may influence the final outcomes. Publication bias arises when studies with positive or significant results are more likely to be disseminated, which can contribute to inconsistencies within the existing literature. Additionally, individual factors like age, gender, and health status significantly influence how participants respond to exercise interventions. For example, certain individuals might see greater improvements with HIIT, while others may experience more favorable outcomes from MICT.

### 4.3 Most effective dose of exercise for obese/overweight adolescents

The 2020 “Guidelines for Physical Activity and Sedentary Behavior” published by the WHO recommends that children and adolescents should perform at least 60 min of moderate-to vigorous-intensity aerobic activity daily. Additionally, guidelines recommend engaging in activities that promote muscle and bone strength at least three times per week (World Health Organization, 2020). A recent Bayesian network meta-analysis highlighted that for adolescents without severe chronic conditions, a daily HIIT dose of approximately 76 METs-minutes may result in clinically meaningful reductions in BMI. In comparison, other forms of exercise typically require around 200 METs-minutes/day to produce similar effects (Huang et al., 2025). Considering the reality that adolescents spend most of their time in school, many studies have incorporated the school environment into their recommendations. Espinoza-Silva et al. suggested that school-age children (7–9 years old) in a school setting engage in a structured HIIT program consisting of 10 rounds of 20-s high-intensity sprints with 10-s breaks. This form of HIIT, which combines jumping, aerobics, and sprints, has been demonstrated to enhance anthropometric and cardiovascular parameters, while also contributing to a reduction in obesity rates among school-age children (Espinoza-Silva et al., 2019). Similarly, Deng and Wang (2024) evaluated 18 RCTs and concluded that HIIT three times per week for overweight/obese children between the ages of 7 and 9 years, involving 2-8 sets of high-intensity exercises that follow a 1:1 work-to-rest ratio, represents the most effective strategy for enhancing anthropometric and cardiovascular health while reducing obesity prevalence in this age group.

Drawing on the results of our meta-analysis and taking into account the practical considerations for adolescents, we suggest that HIIT can positively impact body composition and blood pressure in adolescents aged 10–19 years when performed in shorter, more distributed sessions. From a practical perspective, and incorporating recommendations from all included literature, running and other forms of HIIT can be incorporated into adolescents’ exercise routines at least three times per week, with more than six sessions per session and a work-to-rest ratio of approximately 1:1. High-intensity exercise periods with an intensity greater than or equal to 100% of average daily exercise time (MAS) and recovery periods with an intensity of 50% of MAS appear to be particularly effective in promoting fat loss, weight control, and improving blood pressure. While these recommendations may provide valuable insights for school-based or community-based interventions, further research is needed to refine optimal exercise prescriptions for different adolescent subgroups.

From a practical perspective, the successful implementation of exercise interventions in adolescents depends not only on physiological efficacy but also on adherence, safety, and feasibility. The included studies suggest that shorter, structured HIIT sessions integrated into school routines are generally well-tolerated and maintain good adherence among adolescents (Espinoza-Silva et al., 2019; Deng and Wang, 2024). Safety considerations, including appropriate warm-up, work-to-rest ratios, and gradual progression of intensity, are essential to minimize injury risk and ensure exercise is suitable for overweight/obese youth (Baltich et al., 2017; Costigan et al., 2015). Moreover, feasibility is enhanced when interventions require minimal equipment, can be performed within limited time frames, and are adaptable to school or community settings (Huang et al., 2025; Espinoza-Silva et al., 2019; Deng and Wang, 2024). These practical aspects are crucial for translating exercise prescriptions into real-world settings and should be considered alongside the observed physiological benefits.

### 4.4 Limitations

This study has several limitations, including the following: (1) The overall sample size in the included research is generally small, and there is an absence of large-scale RCTs; (2) Many of the studies did not clearly describe the allocation concealment or blinding methods, which could introduce potential bias; (3) The exercise protocols among the included studies differed with respect to exercise intensity, rest intervals, and duration, which could affect the consistency and precision in relation to the observed results. The dose-dependent effects of exercise characteristics (intensity, frequency, duration) on intervention results should be a focus of future prospective research; (4) As these subgroup analyses were conducted *post hoc*, the findings should be interpreted with caution and require confirmation by future pre-specified studies. Additionally, studies should account for individual factors, including age, gender, and baseline BMI, to provide more tailored intervention recommendations for diverse populations.

## 5 Conclusion

In conclusion, our meta-analysis incorporating 16 RCTs with a combined sample of 473 adolescents classified as overweight or obese suggested that HIIT may provide a modest advantage over MICT in reducing BF% among adolescents aged 10–19 years, although the statistical significance was borderline. Nevertheless, no significant differences were observed between the two exercise modalities in body weight, BMI, FFM, WC, SBP, and DBP, except for a subgroup finding indicating that MICT was more effective in reducing WC when running was used as the intervention mode. Given the limited number of studies, the modest effect size, and the variability in exercise prescriptions, further high-quality RCTs are warranted to confirm these findings and to establish more precise exercise recommendations for this population.

## Data Availability

The original contributions presented in the study are included in the article/Supplementary Material, further inquiries can be directed to the corresponding author.

## References

[B1] AcoS M. (2018). ACSM's guidelines for exercise testing and prescription. 10th ed. Philadelphia, PA: Wolters Kluwer.

[B2] Ardern C. L. Büttner F. Andrade R. Weir A. Ashe M. C. Holden S. (2022). Implementing the 27 PRISMA 2020 statement items for systematic reviews in the sport and exercise medicine, musculoskeletal rehabilitation and sports science fields: the PERSiST (implementing prisma in exercise, rehabilitation, sport medicine and SporTs science) guidance. Br. J. Sports Med. 56 (4), 175–195. 10.1136/bjsports-2021-103987 34625401 PMC8862073

[B3] Asia WHOROfS-E (2021). Adolescent health: world health organization regional office for south-east Asia. Available online at: https://www.who.int/southeastasia/health-topics/adolescent-health.

[B4] Aslankeser Z. Balcı Ş. S. (2017). Re-examination of the contribution of substrates to energy expenditure during high-intensity intermittent exercise in endurance athletes. PeerJ 5, e3769. 10.7717/peerj.3769 28894645 PMC5591632

[B5] Atakan M. M. K. Ş. Güzel Y. Tin H. T. Yan X. (2021). The role of exercise, diet, and cytokines in preventing obesity and improving adipose tissue. Nutrients 13 (5), 1459. 10.3390/nu13051459 33922998 PMC8145589

[B6] Bahreini A. Akbarpour M. Fathi R. Goldashti H. (2021). Evaluation of changes in insulin resistance and serum cortisol levels after eight weeks of continuous and interval aerobic training in healthy and Obese girls. Med. Laboratory J. 15 (3), 33–39. 10.61186/mlj.15.3.33

[B7] Bailey R. C. Olson J. Pepper S. L. Porszasz J. Barstow T. J. Cooper D. M. (1995). The level and tempo of children's physical activities: an observational study. Med. Sci. Sports Exerc 27 (7), 1033–1041. 10.1249/00005768-199507000-00012 7564970

[B8] Baltich J. Emery C. A. Whittaker J. L. Nigg B. M. (2017). Running injuries in novice runners enrolled in different training interventions: a pilot randomized controlled trial. Scand. J. Med. Sci. Sports 27 (11), 1372–1383. 10.1111/sms.12743 27486011

[B9] Baquet G. Gamelin F. X. Aucouturier J. Berthoin S. (2017). Cardiorespiratory responses to continuous and intermittent exercises in children. Int. J. Sports Med. 38 (10), 755–762. 10.1055/s-0043-111892 28783846

[B10] Boutcher S. H. (2011). High-intensity intermittent exercise and fat loss. J. Obes. 2011, 868305. 10.1155/2011/868305 21113312 PMC2991639

[B11] Braun B. Sharoff C. Chipkin S. R. Beaudoin F. (1985)2004). Effects of insulin resistance on substrate utilization during exercise in overweight women. J. Appl. Physiol. 97 (3), 991–997. 10.1152/japplphysiol.00231.2004 15133003

[B12] Bray G. A. Kim K. K. Wilding J. P. H. World O. F. (2017). Obesity: a chronic relapsing progressive disease process. A position statement of the world obesity Federation. Obes. Rev. 18 (7), 715–723. 10.1111/obr.12551 28489290

[B13] Bruce C. R. Thrush A. B. Mertz V. A. Bezaire V. Chabowski A. Heigenhauser G. J. (2006). Endurance training in Obese humans improves glucose tolerance and mitochondrial fatty acid oxidation and alters muscle lipid content. Am. J. Physiol. Endocrinol. Metab. 291 (1), E99–e107. 10.1152/ajpendo.00587.2005 16464906

[B14] Cao M. Tang Y. Li S. Zou Y. (2021). Effects of high-intensity interval training and moderate-intensity continuous training on cardiometabolic risk factors in overweight and obesity children and adolescents: a meta-analysis of randomized controlled trials. Int. J. Environ. Res. Public Health 18 (22), 11905. 10.3390/ijerph182211905 34831659 PMC8623248

[B15] Cao M. Tang Y. C. Li S. Zou Y. (2022). Effects of high-intensity interval training on body composition, cardiopulmonary fitness, and blood markers in Obese children. Chin. J. Sports Med. 41 (02), 109–117.

[B16] Chawla S. Tessarolo Silva F. Amaral Medeiros S. Mekary R. A. Radenkovic D. (2020). The effect of low-fat and low-carbohydrate diets on weight loss and lipid levels: a systematic review and meta-analysis. Nutrients 12 (12), 3774. 10.3390/nu12123774 33317019 PMC7763365

[B17] Cheema B. S. Davies T. B. Stewart M. Papalia S. Atlantis E. (2015). The feasibility and effectiveness of high-intensity boxing training versus moderate-intensity brisk walking in adults with abdominal obesity: a pilot study. BMC Sports Sci. Med. Rehabil. 7, 3. 10.1186/2052-1847-7-3 25973207 PMC4429464

[B18] Christoforidis A. Maniadaki I. Stanhope R. (2005). Growth hormone/insulin-like growth factor-1 axis during puberty. Pediatr. Endocrinol. Rev. 3 (1), 5–10. 16369208

[B19] Cornelissen V. A. Smart N. A. (2013). Exercise training for blood pressure: a systematic review and meta-analysis. J. Am. Heart Assoc. 2 (1), e004473. 10.1161/JAHA.112.004473 23525435 PMC3603230

[B20] Corte de Araujo A. C. Roschel H. Picanço A. R. do Prado D. M. L. Villares S. M. F. de Sá Pinto A. L. (2012). Similar health benefits of endurance and high-intensity interval training in Obese children. PloS one 7 (8), e42747. 10.1371/journal.pone.0042747 22880097 PMC3412799

[B21] Costigan S. A. Eather N. Plotnikoff R. C. Taaffe D. R. Lubans D. R. (2015). High-intensity interval training for improving health-related fitness in adolescents: a systematic review and meta-analysis. Br. J. Sports Med. 49 (19), 1253–1261. 10.1136/bjsports-2014-094490 26089322

[B22] Cumpston M. Li T. Page M. J. Chandler J. Welch V. A. Higgins J. P. (2019). Updated guidance for trusted systematic reviews: a new edition of the Cochrane Handbook for Systematic reviews of Interventions. Cochrane Database Syst. Rev. 10 (10), Ed000142. 10.1002/14651858.ED000142 31643080 PMC10284251

[B23] Cvetkovic N. Stojanovic E. Stojiljkovic N. Nikolic D. Scanlan A. T. Milanovic Z. (2018). Exercise training in overweight and Obese children: recreational football and high-intensity interval training provide similar benefits to physical fitness. Scand. J. Med. Sci. Sports 28 (Suppl. 1), 18–32. 10.1111/sms.13241 29979479

[B24] Da Costa Santos C. M. de Mattos Pimenta C. A. Nobre M. R. (2007). The PICO strategy for the research question construction and evidence search. Rev. Lat. Am. Enferm. 15 (3), 508–511. 10.1590/s0104-11692007000300023 17653438

[B25] Davis J. N. Hodges V. A. Gillham M. B. (2006). Physical activity compliance: differences between overweight/Obese and normal-weight adults. Obesity 14 (12), 2259–2265. 10.1038/oby.2006.265 17189554

[B26] Deng Y. Wang X. (2024). Effect of high-intensity interval training on cardiorespiratory in children and adolescents with overweight or obesity: a meta-analysis of randomized controlled trials. Front. Public Health 12, 1269508. 10.3389/fpubh.2024.1269508 38344230 PMC10853929

[B27] Dias K. A. Ingul C. B. Tjønna A. E. Keating S. E. Gomersall S. R. Follestad T. (2018). Effect of high-intensity interval training on Fitness, fat mass and cardiometabolic biomarkers in children with obesity: a randomised controlled trial. Sports Med. 48 (3), 733–746. 10.1007/s40279-017-0777-0 28853029

[B28] Espinoza-Silva M. Latorre-Román P. Párraga-Montilla J. Caamaño-Navarrete F. Jerez-Mayorga D. Delgado-Floody P. (2019). Response of obese schoolchildren to high-intensity interval training applied in the school context. Endocrinol. Diabetes Nutr. Engl. Ed. 66 (10), 611–619. 10.1016/j.endinu.2019.05.005 31439501

[B29] Fernandez A. C. Túlio de Mello M. Tufik S. Morcelli de Castro P. Fisberg M. (2004). Influence of the aerobic and anaerobic training on the body fat mass in obese adolescents. Rev. Bras. Med. do Esporte 10 (3), 152–164. 10.1590/S1517-86922004000300004

[B30] Frandsen J. Beck T. Langkilde C. H. Larsen S. Dela F. Helge J. W. (2021). The training induced increase in whole-body peak fat oxidation rate May be attenuated with aging. Eur. J. Sport Sci. 21 (1), 69–76. 10.1080/17461391.2020.1721563 31973646

[B31] Freedman D. S. Mei Z. Srinivasan S. R. Berenson G. S. Dietz W. H. (2007). Cardiovascular risk factors and excess adiposity among overweight children and adolescents: the Bogalusa heart study. J. Pediatr. 150 (1), 12–7.e2. 10.1016/j.jpeds.2006.08.042 17188605

[B32] Fryar Cdc T. C. Li X. (2012) “Prevalence of uncontrolled risk factors for cardiovascular disease: united States, 1999–2010,”. Hyattsville, MD. 23101933

[B33] Garber C. E. Blissmer B. Deschenes M. R. Franklin B. A. Lamonte M. J. Lee I. M. (2011). Quantity and quality of exercise for developing and maintaining cardiorespiratory, musculoskeletal, and neuromotor fitness in apparently healthy adults: guidance for prescribing exercise. Med. and Sci. Sports and Exerc. 43 (7), 1334–1359. 10.1249/mss.0b013e318213fefb 21694556

[B34] García-Hermoso A. Cerrillo-Urbina A. J. Herrera-Valenzuela T. Cristi-Montero C. Saavedra J. M. Martínez-Vizcaíno V. (2016). Is high-intensity interval training more effective on improving cardiometabolic risk and aerobic capacity than other forms of exercise in overweight and obese youth? A meta-analysis. Obes. Rev. 17 (6), 531–540. 10.1111/obr.12395 26948135

[B35] Gibala M. J. Little J. P. Macdonald M. J. Hawley J. A. (2012). Physiological adaptations to low-volume, high-intensity interval training in health and disease. J. Physiol. 590 (5), 1077–1084. 10.1113/jphysiol.2011.224725 22289907 PMC3381816

[B36] Gonçalves C. Raimundo A. Abreu A. Pais J. Bravo J. (2024). Effects of high-intensity interval training vs moderate-intensity continuous training on body composition and blood biomarkers in coronary artery disease patients: a randomized controlled trial. Rev. Cardiovasc Med. 25 (3), 102. 10.31083/j.rcm2503102 39076951 PMC11263861

[B37] Guerrini Usubini A. Bottacchi M. Bondesan A. Caroli D. Castelnuovo G. Sartorio A. (2022). A three-week in-hospital multidisciplinary body weight reduction program exerts beneficial effects on physical and mental health and fatiguability of elderly patients with obesity. Front. Aging Neurosci. 14, 1054941. 10.3389/fnagi.2022.1054941 36589548 PMC9800933

[B74] Hansen D. Dendale P. Coninx K. Vanhees L. Piepoli M. F. Niebauer J. (2017). The european association of preventive cardiology exercise prescription in everyday practice and rehabilitative training (EXPERT) tool: a digital training and decision support system for optimized exercise prescription in cardiovascular disease. Concept, definitions and construction methodology. Eur. J. Preventive Cardiology 24 (10), 1017–1031. 10.1177/2047487317702042 28420250

[B38] Henriksson H. Henriksson P. Tynelius P. Ekstedt M. Berglind D. Labayen I. (2019). Cardiorespiratory fitness, muscular strength, and obesity in adolescence and later chronic disability due to cardiovascular disease: a cohort study of 1 million men. Eur. Heart J. 41 (15), 1503–1510. 10.1093/eurheartj/ehz774 31710669 PMC7154806

[B39] Hetlelid K. J. Plews D. J. Herold E. Laursen P. B. Seiler S. (2015). Rethinking the role of fat oxidation: substrate utilisation during high-intensity interval training in well-trained and recreationally trained runners. BMJ Open Sport Exerc Med. 1 (1), e000047. 10.1136/bmjsem-2015-000047 27900134 PMC5117036

[B40] Higgins J. (2008). Cochrane handbook for systematic reviews of interventions. Available online at: http://wwwcochrane-handbookorg.

[B41] Higgins J. P. Thompson S. G. Deeks J. J. Altman D. G. (2003). Measuring inconsistency in meta-analyses. Bmj 327 (7414), 557–560. 10.1136/bmj.327.7414.557 12958120 PMC192859

[B42] Higgins J. P. T. T. J. Chandler J. Cumpston M. Li T. Page M. J. Welch V. A. (2024). Cochrane handbook for systematic reviews of interventions. London: The Cochrane Collaboration.

[B43] Højlund K. Mogensen M. Sahlin K. Beck-Nielsen H. (2008). Mitochondrial dysfunction in type 2 diabetes and obesity. Endocrinol. Metab. Clin. North Am. 37 (3), 713–731. 10.1016/j.ecl.2008.06.006 18775360

[B44] Hooper L. Abdelhamid A. Moore H. J. Douthwaite W. Skeaff C. M. Summerbell C. D. (2012). Effect of reducing total fat intake on body weight: systematic review and meta-analysis of randomised controlled trials and cohort studies. Bmj 345, e7666. 10.1136/bmj.e7666 23220130 PMC3516671

[B45] Hosseini A. M. Chen Y.-F. Jagielski A. Mannan Choudhury S. Banerjee D. Thomas G. N. (2012). Weight loss intervention through lifestyle modification or pharmacotherapy for obstructive sleep apnoea in adults. Cochrane Database Syst. Rev. 10.1002/14651858.CD010281

[B46] Huang Z. Sun G. Li J. Zhang B. Lai G. Jing H. (2025). Optimal exercise dose on body mass index (BMI) in children and adolescents with overweight and obesity: a systematic review and bayesian model-based network meta-analysis. BMC Public Health 25 (1), 215. 10.1186/s12889-025-21405-3 39827114 PMC11742208

[B47] Incognito A. V. Duplea S. G. Lee J. B. Sussman J. Shepherd A. D. Doherty C. J. (2019). Arterial baroreflex regulation of muscle sympathetic nerve activity at rest and during stress. J. Physiol. 597 (18), 4729–4741. 10.1113/JP278376 31368530

[B48] Ingul C. B. Dias K. A. Tjonna A. E. Follestad T. Hosseini M. S. Timilsina A. S. (2018). Effect of high intensity interval training on cardiac function in children with obesity: a randomised controlled trial. Prog. Cardiovasc Dis. 61 (2), 214–221. 10.1016/j.pcad.2018.01.012 29452134

[B49] Islam H. Townsend L. K. Hazell T. J. (2018). Excess postexercise oxygen consumption and fat utilization following submaximal continuous and supramaximal interval running. Res. Q. Exerc Sport 89 (4), 450–456. 10.1080/02701367.2018.1513633 30325710

[B50] Jackson W. F. (2021). Myogenic tone in peripheral resistance arteries and arterioles: the pressure is on. Front. Physiol. 12, 699517. 10.3389/fphys.2021.699517 34366889 PMC8339585

[B51] Jiang L. Zhang Y. Wang Z. Wang Y. (2024). Acute interval running induces greater excess post-exercise oxygen consumption and lipid oxidation than isocaloric continuous running in men with obesity. Sci. Rep. 14 (1), 9178. 10.1038/s41598-024-59893-9 38649759 PMC11035584

[B52] Johansson K. Neovius M. Hemmingsson E. (2014). Effects of anti-obesity drugs, diet, and exercise on weight-loss maintenance after a very-low-calorie diet or low-calorie diet: a systematic review and meta-analysis of randomized controlled trials. Am. J. Clin. Nutr. 99 (1), 14–23. 10.3945/ajcn.113.070052 24172297 PMC3862452

[B53] Julian V. Costa D. O'Malley G. Metz L. Fillon A. Miguet M. (2022). Bone response to high-intensity interval training versus moderate-intensity continuous training in adolescents with obesity. Obes. facts 15 (1), 46–54. 10.1159/000519271 34864737 PMC8820153

[B54] Keating S. E. Johnson N. A. Mielke G. I. Coombes J. S. (2017). A systematic review and meta-analysis of interval training versus moderate-intensity continuous training on body adiposity. Obes. Rev. 18 (8), 943–964. 10.1111/obr.12536 28513103

[B55] Koubaa A. Trabelsi H. Masmoudi L. Elloumi M. Sahnoun Z. Zeghal K. M. (2013). Effect of intermittent and continuous training on body composition cardiorespiratory fitness and lipid profile in Obese adolescents. Iosr J. Of Pharm. 3, 31–37. 10.9790/3013-32103137

[B56] Kwak H. B. (2013). Exercise and obesity-induced insulin resistance in skeletal muscle. Integr. Med. Res. 2 (4), 131–138. 10.1016/j.imr.2013.09.004 28664064 PMC5481720

[B57] Lazzer S. Tringali G. Caccavale M. De Micheli R. Abbruzzese L. Sartorio A. (2017). Effects of high-intensity interval training on physical capacities and substrate oxidation rate in obese adolescents. J. Endocrinol. Invest 40 (2), 217–226. 10.1007/s40618-016-0551-4 27639403

[B58] Leite N. Pizzi J. de Menezes Junior F. J. Tadiotto M. C. de Jesus I. C. Corazza P. R. P. (2022). Effect of mict and hiit on cardiometabolic risk and body composition in obese boys. Rev. Bras. Med. do esporte 28 (4), 274–280. 10.1590/1517-8692202228042020_0129

[B59] Li S. Cao M. Zou Y. Tang Y. C. (2023). Effects of high-intensity interval training on visceral fat and cardiopulmonary fitness in obese children. J. Phys. Educ. 30 (04), 138–144. 10.16237/j.cnki.cn44-1404/g8

[B60] Lobstein T. Jackson-Leach R. Moodie M. L. Hall K. D. Gortmaker S. L. Swinburn B. A. (2015). Child and adolescent obesity: part of a bigger picture. Lancet 385 (9986), 2510–2520. 10.1016/s0140-6736(14)61746-3 25703114 PMC4594797

[B61] Luo P. Wu R. Gao W. Yan W. Wang R. Ye Y. (2024). Effects of high-intensity interval exercise on arterial stiffness in individuals at risk for cardiovascular disease: a meta-analysis. Front. Cardiovasc. Med. 11–2024. 10.3389/fcvm.2024.1376861 38694567 PMC11061535

[B62] Mancuso P. Bouchard B. (2019). The impact of aging on adipose function and adipokine synthesis. Front. Endocrinol. (Lausanne) 10, 137. 10.3389/fendo.2019.00137 30915034 PMC6421296

[B63] Meng C. Yucheng T. Shu L. Yu Z. (2022). Effects of school-based high-intensity interval training on body composition, cardiorespiratory fitness and cardiometabolic markers in adolescent boys with obesity: a randomized controlled trial. BMC Pediatr. 22 (1), 112. 10.1186/s12887-021-03079-z 35232402 PMC8886768

[B64] Miguet M. Fearnbach N. S. Metz L. Khammassi M. Julian V. Cardenoux C. (2020). Effect of HIIT versus MICT on body composition and energy intake in dietary restrained and unrestrained adolescents with obesity. Appl. Physiol. Nutr. Metab. 45 (4), 437–445. 10.1139/apnm-2019-0160 31505120

[B65] Miles L. (2007). Physical activity and health. Nutr. Bull. 32 (4), 314–363. 10.1111/j.1467-3010.2007.00668.x

[B66] Moher D. Liberati A. Tetzlaff J. Altman D. G. PRISMA Group (2009). Preferred reporting items for systematic reviews and meta-analyses: the PRISMA statement. PLoS Med. 6 (7), e1000097. 10.1371/journal.pmed.1000097 19621072 PMC2707599

[B67] Morrissey C. Montero D. Raverdy C. Masson D. Amiot M. J. Vinet A. (2018). Effects of exercise intensity on microvascular function in obese adolescents. Int. J. Sports Med. 39, 450–455. 10.1055/a-0577-4280 29710370

[B68] Neeland I. J. Ross R. Després J.-P. Matsuzawa Y. Yamashita S. Shai I. (2019). Visceral and ectopic fat, atherosclerosis, and cardiometabolic disease: a position statement. Lancet Diabetes and Endocrinology 7 (9), 715–725. 10.1016/S2213-8587(19)30084-1 31301983

[B69] Norton K. Norton L. Sadgrove D. (2010). Position statement on physical activity and exercise intensity terminology. J. Sci. Med. Sport 13 (5), 496–502. 10.1016/j.jsams.2009.09.008 20005170

[B70] O'Driscoll J. M. Wright S. M. Taylor K. A. Coleman D. A. Sharma R. Wiles J. D. (1985)2018). Cardiac autonomic and left ventricular mechanics following high intensity interval training: a randomized crossover controlled study. J. Appl. Physiol. 125 (4), 1030–1040. 10.1152/japplphysiol.00056.2018 29952247 PMC6230570

[B71] Ortega F. B. Ruiz J. R. Castillo M. J. Sjöström M. (2008). Physical fitness in childhood and adolescence: a powerful marker of health. Int. J. Obes. (Lond). 32 (1), 1–11. 10.1038/sj.ijo.0803774 18043605

[B72] Osawa Y. Azuma K. Tabata S. Katsukawa F. Ishida H. Oguma Y. (2014). Effects of 16-week high-intensity interval training using upper and lower body ergometers on aerobic fitness and morphological changes in healthy men: a preliminary study. Open Access J. Sports Med. 5, 257–265. 10.2147/OAJSM.S68932 25395872 PMC4226445

[B73] Page M. J. McKenzie J. E. Bossuyt P. M. Boutron I. Hoffmann T. C. Mulrow C. D. (2021). The PRISMA 2020 statement: an updated guideline for reporting systematic reviews. Bmj 372, n71. 10.1136/bmj.n71 33782057 PMC8005924

[B75] Peake J. M. T. S. Markworth J. F. Broadbent J. A. Skinner T. L. Cameron-Smith D. (2014). Metabolic and hormonal responses to isoenergetic high-intensity interval exercise and continuous moderate-intensity exercise. Am. J. Physiology-Endocrinology Metabolism 307 (7), E539–E552. 10.1152/ajpendo.00276.2014 25096178

[B76] Poon E. T. Li H. Y. Little J. P. Wong S. H. Ho R. S. (2024). Efficacy of interval training in improving body composition and adiposity in apparently healthy adults: an umbrella review with meta-analysis. Sports Med. 54 (11), 2817–2840. 10.1007/s40279-024-02070-9 39003682 PMC11560999

[B77] Safar M. E. (2004). Peripheral pulse pressure, large arteries, and microvessels. Hypertension 44 (2), 121–122. 10.1161/01.HYP.0000135448.73199.75 15210654

[B78] Savović J. Jones H. E. Altman D. G. Harris R. J. Jüni P. Pildal J. (2012). Influence of reported study design characteristics on intervention effect estimates from randomized, controlled trials. Ann. Intern Med. 157 (6), 429–438. 10.7326/0003-4819-157-6-201209180-00537 22945832

[B79] Se B. Expert Committee (2007). Expert committee recommendations regarding the prevention, assessment, and treatment of child and adolescent overweight and obesity: summary report. Pediatrics 120 (Suppl. 4), S164–S192. 10.1542/peds.2007-2329C 18055651

[B80] Seravalle G. Mancia G. Grassi G. (2018). Sympathetic nervous system, sleep, and hypertension. Curr. Hypertens. Rep. 20 (9), 74. 10.1007/s11906-018-0874-y 29980938

[B81] Shahoud Jss T. Aeddula N. R. P. (2023). Arterial pressure regulation. StatPearls. Treasure Island (FL): StatPearls Publishing. 30860744

[B82] Shi W. X. Chen J. A. He Y. F. Su P. Wang M. Y. Li X. L. (2022). The effects of high-intensity interval training and moderate-intensity continuous training on visceral fat and carotid hemodynamics parameters in obese adults. J. Exerc. Sci. and Fit. 20 (4), 355–365. 10.1016/j.jesf.2022.09.001 36186829 PMC9486563

[B83] Sijie T. Hainai Y. Fengying Y. Jianxiong W. (2012). High intensity interval exercise training in overweight young women. J. Sports Med. Phys. Fit. 52 (3), 255–262. 22648463

[B84] Sim A. Y. Wallman K. E. Fairchild T. J. Guelfi K. J. (2015). Effects of high-intensity intermittent exercise training on appetite regulation. Med. Sci. Sports Exerc 47 (11), 2441–2449. 10.1249/MSS.0000000000000687 25899101

[B85] Skinner A. C. Perrin E. M. Moss L. A. Skelton J. A. (2015). Cardiometabolic risks and severity of obesity in children and young adults. N. Engl. J. Med. 373 (14), 1307–1317. 10.1056/NEJMoa1502821 26422721

[B86] Starkoff B. E. Eneli I. U. Bonny A. E. Hoffman R. P. Devor S. T. (2014). Estimated aerobic capacity changes in adolescents with obesity following high intensity interval exercise. 2(3) 8.

[B87] Stefanakis K. Kokkorakis M. Mantzoros C. S. (2024). The impact of weight loss on fat-free mass, muscle, bone and hematopoiesis health: implications for emerging pharmacotherapies aiming at fat reduction and lean mass preservation. Metabolism 161, 156057. 10.1016/j.metabol.2024.156057 39481534

[B88] Su Z. Y. Yu W. L. Yan Z. W. Ding D. D. Fang C. C. Luo Q. L. (2024). Comparison of high-intensity interval training and moderate-intensity continuous training on cardiopulmonary function, cardiac autonomic function and vascular function in adolescent boys with obesity: a randomized controlled trial. Eur. J. Sport Sci. 24 (12), 1871–1882. 10.1002/ejsc.12207 39500636 PMC11621380

[B89] Sultana R. N. Sabag A. Keating S. E. Johnson N. A. (2019). The effect of low-volume high-intensity interval training on body composition and cardiorespiratory fitness: a systematic review and meta-analysis. Sports Med. 49 (11), 1687–1721. 10.1007/s40279-019-01167-w 31401727

[B90] Taylor R. S. Ashton K. E. Moxham T. Hooper L. Ebrahim S. (2011). Reduced dietary salt for the prevention of cardiovascular disease. Cochrane Database Syst. Rev. (7), Cd009217. 10.1002/14651858.CD009217 21735439 PMC4160847

[B91] Urbina E. M. Gao Z. Khoury P. R. Martin L. J. Dolan L. M. (2012). Insulin resistance and arterial stiffness in healthy adolescents and young adults. Diabetologia 55 (3), 625–631. 10.1007/s00125-011-2412-1 22193511 PMC3269756

[B92] Urbina E. M. Daniels S. R. Sinaiko A. R. (2023). Blood pressure in children in the 21st century: what do we know and where do we Go from here? Hypertension 80 (8), 1572–1579. 10.1161/HYPERTENSIONAHA.122.19455 37278234 PMC10524445

[B93] Wang Y. Wang S. Meng X. Zhou H. (2024). Effect of high-intensity interval training and moderate-intensity continuous training on cardiovascular risk factors in adolescents: systematic review and meta-analysis of randomized controlled trials. Physiol. Behav. 275, 114459. 10.1016/j.physbeh.2024.114459 38190958

[B94] Ward L. C. (2019). Bioelectrical impedance analysis for body composition assessment: reflections on accuracy, clinical utility, and standardisation. Eur. J. Clin. Nutr. 73 (2), 194–199. 10.1038/s41430-018-0335-3 30297760

[B95] Way K. L. Sultana R. N. Sabag A. Baker M. K. Johnson N. A. (2019). The effect of high Intensity interval training versus moderate intensity continuous training on arterial stiffness and 24h blood pressure responses: a systematic review and meta-analysis. J. Sci. Med. Sport 22 (4), 385–391. 10.1016/j.jsams.2018.09.228 30803498

[B96] Wen J. F. Lou K. (2018). Effects of high-intensity interval training on body composition in overweight children aged 10-12 years. J. Fuyang Normal Univ. Sci. 35 (02), 95–7+102. 10.14096/j.cnki.cn34-1069/n/1004-4329(2018)02-095-03

[B97] Weston K. S. W. U. Coombes J. S. (2014). High-intensity interval training in patients with lifestyle-induced cardiometabolic disease: a systematic review and meta-analysis. Br. J. Sports Med. 48 (16), 1227–1234. 10.1136/bjsports-2013-092576 24144531

[B98] Wewege M. van den Berg R. Ward R. E. Keech A. (2017). The effects of high-intensity interval training vs. moderate-intensity continuous training on body composition in overweight and obese adults: a systematic review and meta-analysis. Obes. Rev. 18 (6), 635–646. 10.1111/obr.12532 28401638

[B99] World Health Organization (2019). New WHO-led study says majority of adolescents worldwide are not sufficiently physically active, putting their current and future health at risk 2019. Available online at: https://www.who.int/news/item/22-11-2019-new-who-led-study-says-majority-of-adolescents-worldwide-are-not-sufficiently-physically-active-putting-their-current-and-future-health-at-risk.

[B100] World Health Organization (2020). WHO guidelines on physical activity and sedentary behaviour. Geneva. 10.1136/bjsports-2020-102955 PMC7719906 33239350

[B101] World Health Organization (2021). Obesity and Overweight [Fact Sheet]. Geneva: World Health Organization. Available online at: https://www.who.int/news-room/fact-sheets/detail/obesity-and-overweight.

[B102] World Health Organization (2022). International classification of diseases 11th revision 2022. Available online at: https://icd.who.int/en/.

[B103] World Health Organization (2024a). World obesity day 2024 - obesity and youth: young people catalyzing change. Available online at: https://www.who.int/news-room/events/detail/2024/03/04/default-calendar/world-obesity-day-2024-obesity-youth-young-people-catalyzing-change.

[B104] World Health Organization (2024b). Youth health. Geneva: World Health Organization. Available online at: https://www.who.int/zh/health-topics/adolescent-health#tab=tab_1.

[B105] Zampetti S. Campagna G. Leto G. Lucantoni F. D'Onofrio L. Marandola L. (2018). Relation between wrist circumference and left ventricular structure in overweight children. Am. J. Cardiol. 121 (12), 1624–1628. 10.1016/j.amjcard.2018.02.057 29650237

[B106] Zheng W. Yin M. Guo Y. Liu H. Sun J. Zhu A. (2025). Effects and moderator of high-intensity interval training and moderate-intensity continuous training among children and adolescents with overweight or obese: a systematic review and meta-analysis. Front. Physiol. 16, 1625516. 10.3389/fphys.2025.1625516 40809289 PMC12343602

[B107] Zhou J. Sun W. Tang S. Jiang D. Tan B. Li S. (2025). Effects of exercise interventions on blood pressure in children and adolescents with overweight or obesity: a systematic review and meta-analysis of randomized controlled trials. J. Adolesc. Health 76 (3), 361–369. 10.1016/j.jadohealth.2024.09.017 39864002

