# Supplementary material for: Comparative effects of high-intensity interval training versus moderate-intensity continuous training on body composition and blood pressure in overweight adolescents: a systematic review and meta-analysis of randomized controlled trials
Source: Front Physiol. 2025 Oct 1;16:1636792. doi: 10.3389/fphys.2025.1636792 (PMC12521180; doi:10.3389/fphys.2025.1636792)
Supplement: Supplementary file 1 [file Supplementaryfile1.docx]

Supplementary Material

| **Comparative Effects of High-Intensity Interval Training versus Moderate-Intensity Continuous Training on Body Weight and Blood Pressure in Overweight Adolescents: A Systematic Review and Meta-Analysis of Randomized Controlled Trials** |
| --- |

[**Table S1 Search strategy (Fed 20, 2025) detailed for PubMed** 2](#_Toc197268560)

[**Table S2 Search strategy (Fed 20, 2025) detailed for Web of Science** 3](#_Toc197268561)

[**Table S3. Search strategy (Fed 20, 2025) detailed for EBSCOhost** 3](#_Toc197268562)

[**Table S4. Search strategy (Fed 20, 2025) detailed for Cochrane Central** 4](#_Toc197268563)

[**Table S5. Search strategy (Fed 20, 2025) detailed for CNKI** 4](#_Toc197268564)

[**Table S6. Search strategy (Fed 20, 2025) detailed for Embase** 5](#_Toc197268565)

[**Table S7. Excluded studies by reason for exclusion (N=90)** 5](#_Toc197268566)

[**Figure S1 Risk of bias assessment.** 13](#_Toc197268567)

[**Table S9. PRISMA Abstracts checklist** 14](#_Toc197268568)

[**Table S10. PRISMA checklist.** 15](#_Toc197268569)

**Table S1 Search strategy (Fed 20, 2025) detailed for PubMed**

| **ID** | **Query** | **Results** |
| --- | --- | --- |
| 1 | "Overweight"[Title/Abstract] OR "Obese"[Title/Abstract] OR "Obesity"[Title/Abstract] OR "Fat Mass"[Title/Abstract] OR "Weight"[Title/Abstract] OR "BMI"[Title/Abstract] OR "Body | 2,114,149 |
| 2 | "Adolescent"[Title/Abstract] OR "Teen"[Title/Abstract] OR "Adolescence"[Title/Abstract] OR "Teenager"[Title/Abstract] OR "Teenagers"[Title/Abstract] OR "Youth"[Title/Abstract] OR "Youths"[Title/Abstract] OR "Young People"[Title/Abstract] OR "Pediatric"[Title/Abstract] OR "Children"[Title/Abstract] | 1,817,863 |
| 3 | "MICT"[Title/Abstract] OR "Moderate-Intensity Continuous Training"[Title/Abstract] OR "Endurance Training"[Title/Abstract] OR "Aerobic Training"[Title/Abstract] OR "Moderate-Intensity Exercise"[Title/Abstract] OR "Moderate-Intensity Training"[Title/Abstract] | 12,661 |
| 4 | "HIIT"[Title/Abstract] OR "High-Intensity Interval Training"[Title/Abstract] OR "Interval Training"[Title/Abstract] OR "Intermittent Exercise"[Title/Abstract] OR "Interval Exercise"[Title/Abstract] OR "Intermittent Training"[Title/Abstract] | 7,622 |
| 5 | ((("HIIT"[Title/Abstract] OR "High-Intensity Interval Training"[Title/Abstract] OR "Interval Training"[Title/Abstract] OR "Intermittent Exercise"[Title/Abstract] OR "Interval Exercise"[Title/Abstract] OR "Intermittent Training"[Title/Abstract]) AND ("MICT"[Title/Abstract] OR "Moderate-Intensity Continuous Training"[Title/Abstract] OR "Endurance Training"[Title/Abstract] OR "Aerobic Training"[Title/Abstract] OR "Moderate-Intensity Exercise"[Title/Abstract] OR "Moderate-Intensity Training"[Title/Abstract])) AND ("Adolescent"[Title/Abstract] OR "Teen"[Title/Abstract] OR "Adolescence"[Title/Abstract] OR "Teenager"[Title/Abstract] OR "Teenagers"[Title/Abstract] OR "Youth"[Title/Abstract] OR "Youths"[Title/Abstract] OR "Young People"[Title/Abstract] OR "Pediatric"[Title/Abstract] OR "Children"[Title/Abstract])) AND ("Overweight"[Title/Abstract] OR "Obese"[Title/Abstract] OR "Obesity"[Title/Abstract] OR "Fat Mass"[Title/Abstract] OR "Weight"[Title/Abstract] OR "BMI"[Title/Abstract] OR "Body Composition"[Title/Abstract] OR "Blood Pressure"[Title/Abstract] OR "Hypertension"[Title/Abstract] OR "Systolic Pressure"[Title/Abstract] OR "Diastolic Pressure"[Title/Abstract] OR "Cardiovascular Health"[Title/Abstract]) | 43 |

**Table S2 Search strategy (Fed 20, 2025) detailed for Web of Science**

| **ID** | **Search** | **Results** |
| --- | --- | --- |
| #1 | TS=("HIIT" OR "High-Intensity Interval Training" OR "Interval Training" OR "Intermittent Exercise" OR "Interval Exercise" OR "Intermittent Training") | 8,017 |
| #2 | TS=("MICT" OR "Moderate-Intensity Continuous Training" OR "Endurance Training" OR "Aerobic Training" OR "Moderate-Intensity Exercise" OR "Moderate-Intensity Training") | 9,725 |
| #3 | TS=("Adolescent" OR "Teen" OR "Adolescence" OR "Teenager" OR "Teenagers" OR "Youth" OR "Youths" OR "Young People" OR "Pediatric" OR "Children") | 1,478,253 |
| #4 | TS=("Overweight" OR "Obese" OR "Obesity" OR "Fat Mass" OR "Weight" OR "BMI" OR "Body Composition" OR "Blood Pressure" OR "Hypertension" OR "Systolic Pressure" OR "Diastolic Pressure" OR "Cardiovascular Health") | 1,807,796 |
| #5 | #1 AND #2 AND #3 AND #4 | 59 |

**Table S3. Search strategy (Fed 20, 2025) detailed for EBSCOhost**

| ID | Search | Results |
| --- | --- | --- |
| S1 | ("HIIT" OR "High-Intensity Interval Training" OR "Interval Training" OR "Intermittent Exercise" OR "Interval Exercise" OR "Intermittent Training") | 24,216 |
| S2 | ("MICT" OR "Moderate-Intensity Continuous Training" OR "Endurance Training" OR "Aerobic Training" OR "Moderate-Intensity Exercise" OR "Moderate-Intensity Training") | 30,631 |
| S3 | ("Adolescent" OR "Teen" OR "Adolescence" OR "Teenager" OR "Teenagers" OR "Youth" OR "Youths" OR "Young People" OR "Pediatric" OR "Children") | 11,594,155 |
| S4 | ("Overweight" OR "Obese" OR "Obesity" OR "Fat Mass" OR "Weight" OR "BMI" OR "Body Composition" OR "Blood Pressure" OR "Hypertension" OR "Systolic Pressure" OR "Diastolic Pressure" OR "Cardiovascular Health" OR "SBP" OR "DBP" OR "MBP") | 4,848,467 |
| S5 | ((("HIIT"[Title/Abstract] OR "High-Intensity Interval Training"[Title/Abstract] OR "Interval Training"[Title/Abstract] OR "Intermittent Exercise"[Title/Abstract] OR "Interval Exercise"[Title/Abstract] OR "Intermittent Training"[Title/Abstract]) AND ("MICT"[Title/Abstract] OR "Moderate-Intensity Continuous Training"[Title/Abstract] OR "Endurance Training"[Title/Abstract] OR "Aerobic Training"[Title/Abstract] OR "Moderate-Intensity Exercise"[Title/Abstract] OR "Moderate-Intensity Training"[Title/Abstract])) AND ("Adolescent"[Title/Abstract] OR "Teen"[Title/Abstract] OR "Adolescence"[Title/Abstract] OR "Teenager"[Title/Abstract] OR "Teenagers"[Title/Abstract] OR "Youth"[Title/Abstract] OR "Youths"[Title/Abstract] OR "Young People"[Title/Abstract] OR "Pediatric"[Title/Abstract] OR "Children"[Title/Abstract])) AND ("Overweight"[Title/Abstract] OR "Obese"[Title/Abstract] OR "Obesity"[Title/Abstract] OR "Fat Mass"[Title/Abstract] OR "Weight"[Title/Abstract] OR "BMI"[Title/Abstract] OR "Body Composition"[Title/Abstract] OR "Blood Pressure"[Title/Abstract] OR "Hypertension"[Title/Abstract] OR "Systolic Pressure"[Title/Abstract] OR "Diastolic Pressure"[Title/Abstract] OR "Cardiovascular Health"[Title/Abstract]) | 129 |

**Table S4. Search strategy (Fed 20, 2025) detailed for Cochrane Central**

| ID | Search | Results |
| --- | --- | --- |
| #1 | ('HIIT':ti,ab OR 'High-Intensity Interval Training':ti,ab OR 'Interval Training':ti,ab OR 'Intermittent Exercise':ti,ab OR 'Interval Exercise':ti,ab OR 'Intermittent Training':ti,ab) | 5,560 |
| #2 | ('MICT':ti,ab OR 'Moderate-Intensity Continuous Training':ti,ab OR 'Endurance Training':ti,ab OR 'Aerobic Training':ti,ab OR 'Moderate-Intensity Exercise':ti,ab OR 'Moderate-Intensity Training':ti,ab) | 8,363 |
| #3 | ('Adolescent'/exp OR 'Teen':ti,ab OR 'Adolescence':ti,ab OR 'Teenager':ti,ab OR 'Teenagers':ti,ab OR 'Youth':ti,ab OR 'Youths':ti,ab OR 'Young People':ti,ab OR 'Pediatric':ti,ab OR 'Children':ti,ab) | 320,370 |
| #4 | ('Overweight'/exp OR 'Obese'/exp OR 'Obesity'/exp OR 'Fat Mass':ti,ab OR 'Weight':ti,ab OR 'BMI':ti,ab OR 'Body Composition':ti,ab OR 'Blood Pressure'/exp OR 'Hypertension'/exp OR 'Systolic Pressure':ti,ab OR 'Diastolic Pressure':ti,ab OR 'Cardiovascular Health':ti,ab) | 354,129 |
| #5 | #1 AND #2 AND #3 AND #4 | 106 |

**Table S5. Search strategy (Fed 20, 2025) detailed for CNKI**

| Search | Results |
| --- | --- |
| (HIIT + High-Intensity Interval Training + Interval Training) AND (MICT + Moderate-Intensity Continuous Training + Endurance Training) AND (Adolescent + Child + Youth + Minor + Student) AND (Overweight + Obesity + Weight + Blood Pressure + Composition) | 80 |

**Table S6. Search strategy (Fed 20, 2025) detailed for Embase**

| ID | Search | Results |
| --- | --- | --- |
| #1 | ('HIIT':ti,ab OR 'High-Intensity Interval Training':ti,ab OR 'Interval Training':ti,ab OR 'Intermittent Exercise':ti,ab OR 'Interval Exercise':ti,ab OR 'Intermittent Training':ti,ab) | 8,957 |
| #2 | ('MICT':ti,ab OR 'Moderate-Intensity Continuous Training':ti,ab OR 'Endurance Training':ti,ab OR 'Aerobic Training':ti,ab OR 'Moderate-Intensity Exercise':ti,ab OR 'Moderate-Intensity Training':ti,ab) | 16,105 |
| #3 | ('Adolescent'/exp OR 'Teen':ti,ab OR 'Adolescence':ti,ab OR 'Teenager':ti,ab OR 'Teenagers':ti,ab OR 'Youth':ti,ab OR 'Youths':ti,ab OR 'Young People':ti,ab OR 'Pediatric':ti,ab OR 'Children':ti,ab) | 3,733,586 |
| #4 | ('Overweight'/exp OR 'Obese'/exp OR 'Obesity'/exp OR 'Fat Mass':ti,ab OR 'Weight':ti,ab OR 'BMI':ti,ab OR 'Body Composition':ti,ab OR 'Blood Pressure'/exp OR 'Hypertension'/exp OR 'Systolic Pressure':ti,ab OR 'Diastolic Pressure':ti,ab OR 'Cardiovascular Health':ti,ab) | 3,657,454 |
| #5 | #1 AND #2 AND #3 AND #4 | 73 |

**Table S7. Excluded studies by reason for exclusion (N=79)**

| **Not RCT(n=3)** | |
| --- | --- |
| 1 | Colpitts, B.H., et al., Irisin response to acute moderate intensity exercise and high intensity interval training in youth of different obesity statuses: A randomized crossover trial. Physiol Rep, 2022. 10(4): p. e15198. |
| 2 | de Faria, W.F., et al., Acute Perceptive Responses to 2 Combined Training Methods in Adolescents: A Crossover Study. Pediatric Exercise Science, 2022. 34(3): p. 152-161. |
| 3 | Murphy, A., et al., The feasibility of high-intensity interval exercise in obese adolescents. Clin Pediatr (Phila), 2015. 54(1): p. 87-90. |
| **Full text not found(n=2)** | |
| 1 | Shenoy Basti, A.R., et al., Effect of high-intensity interval training vs. moderate-intensity continuous training on cardiometabolic risk factors in overweight and obese individuals. Journal of Basic & Clinical Physiology & Pharmacology, 2024. 35(4/5): p. 265-271. |
| 2 | Tadiotto, M.C., et al., Effects and individual response of continuous and interval training on adiponectin concentration, cardiometabolic risk factors, and physical fitness in overweight adolescents. European Journal of Pediatrics, 2023. 182(6): p. 2881-2889. |
| **Age does not match(n=29)** | |
| 1 | Liang JY and Hao L, Effects of high-intensity interval exercise on body composition blood pressure and serum Chemerin in obese children. Chinese Journal School Health, 2018. 39(11): p. 1729-1732. |
| 2 | Liu, J., et al., Effects of different exercise modalities on cardiorespiratory fitness and body composition in overweight male youth. Medical Journal of Chinese People’s Armed Police Force, 2023. 34(11): p. 930-934. |
| 3 | Liu J, Hou LL, and Han SK, Improvement of body fat and cardiorespiratory function in overweight young women by two different intensities of indoor exercise. Chinese Journal of Rehabilitation Medicine, 2022. 37(11): p. 1511-1516. |
| 4 | Zhao J, Liang JY, and Hao L. Effects of moderate- to high-intensity exercise on body composition and cardiovascular function indices in obese female college students. Chinese Journal of School Health, 2020. 41(05): p. 751-754. |
| 5 | Arboleda Serna, V.H., et al., Effects of a high-intensity interval training program versus a moderate-intensity continuous training program on maximal oxygen uptake and blood pressure in healthy adults: study protocol for a randomized controlled trial. Trials, 2016. 17: p. 413. |
| 6 | Chin, E.C., et al., Low-Frequency HIIT Improves Body Composition and Aerobic Capacity in Overweight Men. Medicine and science in sports and exercise, 2020. 52(1): p. 56‐66. |
| 7 | Clark, T., et al., High-intensity interval training for reducing blood pressure: a randomized trial vs. moderate-intensity continuous training in males with overweight or obesity. Hypertension research : official journal of the Japanese Society of Hypertension, 2020. 43(5): p. 396-403. |
| 8 | de Oliveira, G.H., et al., The impact of high-intensity interval training (HIIT) and moderate-intensity continuous training (MICT) on arterial stiffness and blood pressure in young obese women: a randomized controlled trial. Hypertension research, 2020. 43(11): p. 1315‐1318. |
| 9 | Enríquez-Schmidt, J., et al., Moderate-intensity constant or high-intensity interval training? Metabolic effects on candidates to undergo bariatric surgery. Nutrition, metabolism, and cardiovascular diseases: NMCD, 2024. 34(7): p. 1681‐1691. |
| 10 | Fisher, G., et al., High Intensity Interval- vs Moderate Intensity- Training for Improving Cardiometabolic Health in Overweight or Obese Males: a Randomized Controlled Trial. PloS one, 2015. 10(10): p. e0138853. |
| 11 | Hu, M., et al., Interval training causes the same exercise enjoyment as moderate-intensity training to improve cardiorespiratory fitness and body composition in young Chinese women with elevated BMI. Journal of sports sciences, 2021. 39(15): p. 1677‐1686. |
| 12 | Jürimäe, J., et al., Changes in irisin, inflammatory cytokines and aerobic capacity in response to three weeks of supervised sprint interval training in older men. Journal of Sports Medicine and Physical Fitness, 2023. 63(1): p. 162-169. |
| 13 | Kong, Z., et al., Affective and Enjoyment Responses to Short-Term High-Intensity Interval Training with Low-Carbohydrate Diet in Overweight Young Women. Nutrients, 2020. 12(2): p. 442. |
| 14 | Kong, Z., et al., Short-Term High-Intensity Interval Training on Body Composition and Blood Glucose in Overweight and Obese Young Women. Journal of diabetes research, 2016. 2016: p. 4073618. |
| 15 | Nie, J., et al., Impact of high-intensity interval training and moderate-intensity continuous training on resting and postexercise cardiac troponin T concentration. Experimental physiology, 2018. 103(3): p. 370‐380. |
| 16 | Sawyer, B.J., et al., Effects of high-intensity interval training and moderate-intensity continuous training on endothelial function and cardiometabolic risk markers in obese adults. Journal of applied physiology (Bethesda, Md. : 1985), 2016. 121(1): p. 279‐288. |
| 17 | Shi, W.X., et al., The effects of high-intensity interval training and moderate-intensity continuous training on visceral fat and carotid hemodynamics parameters in obese adults. Journal of Exercise Science & Fitness, 2022. 20(4): p. 355-365. |
| 18 | Song, X., et al., Comparative effects of high-intensity interval training and moderate-intensity continuous training on weight and metabolic health in college students with obesity. Scientific reports, 2024. 14(1): p. 16558. |
| 19 | Su, Z.Y., et al., Comparison of high-intensity interval training and moderate-intensity continuous training on cardiopulmonary function, cardiac autonomic function and vascular function in adolescent boys with obesity: A randomized controlled trial. Eur J Sport Sci, 2024. 24(12): p. 1871-1882. |
| 20 | Sun, S., et al., Non-Energy-Restricted Low-Carbohydrate Diet Combined with Exercise Intervention Improved Cardiometabolic Health in Overweight Chinese Females. Nutrients, 2019. 11(12). |
| 21 | Vella, C.A., K. Taylor, and D. Drummer, High-intensity interval and moderate-intensity continuous training elicit similar enjoyment and adherence levels in overweight and obese adults. European journal of sport science, 2017. 17(9): p. 1203‐1211. |
| 22 | Zhang, H., et al., *Exercise training-induced visceral fat loss in obese women: the role of training intensity and modality.* Scandinavian journal of medicine & science in sports, 2021. **31**(1): p. 30‐43. |
| 23 | Zhang, H., et al., *Comparable Effects of High-Intensity Interval Training and Prolonged Continuous Exercise Training on Abdominal Visceral Fat Reduction in Obese Young Women.* Journal of diabetes research, 2017. **2017**: p. 5071740. |
| 24 | Chen Shan and Song Guangxian, Effects of high-intensity interval training on body morphometric indices in overweight young women. Sports Horizons, 2024(20): p. 95-97. |
| 25 | Jiang, L., et al., The Correlation Between Insulin Resistance and Blood Lipids in Children. Journal of Medical Biochemistry, 2024. 43(6): p. 860-869. |
| 26 | Li, J., et al., The Correlation Between Insulin Resistance and Blood Lipids in Children. Journal of Medical Biochemistry, 2024. 43(6): p. 860-869. |
| 27 | Martino, S.A., et al., The effect of exercise intensity on cardiovascular health in children who are overweight or obese: two pilot studies. Cardiopulmonary physical therapy journal, 2022. 33(1): p. e15‐e16. |
| 28 | Zuo, C., et al., School-based high-intensity interval exercise program in children with overweight induce a greater improvements in body composition and physical fitness than moderate-intensity continuous exercise. BMC public health, 2023. 23(1): p. 2210. |
| **Subjects not overweight(n=32)** | |
| 1 | Wang, Renzhong, Wang, Zhanbei, and Lu, Qing, An experimental study of the effects of HIIT and MICT on endurance and cardiorespiratory fitness in middle school students. Physical Education Review, 2023. 42(07): p. 82-84+88. |
| 2 | Ahmad, F., et al., Enhancing Selective Attention: A Comparative Study of Moderate-Intensity Exercise and High-Intensity Interval Exercise in Young Adults. Journal of Medical Sciences (1997-3438), 2024. 32(3): p. 255-259. |
| 3 | Belamjahad, A., et al., Effects of a Preseason Neuromuscular Training Program vs. an Endurance-Dominated Program on Physical Fitness and Injury Prevention in Female Soccer Players. Sports medicine - open, 2024. 10(1). |
| 4 | Berglund, I., et al., The Long-term Effect of Different Exercise Intensities on High-Density Lipoprotein Cholesterol in Older Men and Women Using the Per Protocol Approach: The Generation 100 Study. Mayo Clinic proceedings. Innovations, quality & outcomes, 2021. 5(5): p. 859-871. |
| 5 | Bluett, K.A., M.B.A. De Ste Croix, and R.S. Lloyd, A preliminary investigation into concurrent aerobic and resistance training in youth runners. Isokinetics and Exercise Science, 2015. 23(2): p. 77-85. |
| 6 | Bond, B., et al., Exercise intensity and postprandial health outcomes in adolescents. European Journal of Applied Physiology, 2015. 115(5): p. 927-936. |
| 7 | Bond, B., et al., Accumulating exercise and postprandial health in adolescents. Metabolism, 2015. 64(9): p. 1068-76. |
| 8 | Camacho-Cardenosa, A., et al., Effects of High Intensity Interval Training on Fat Mass Parameters in Adolescents. Rev Esp Salud Publica, 2016. 90: p. e1-e9. |
| 9 | Chamari, K., et al., Effects of aging on cardiorespiratory responses to brief and intense intermittent exercise in endurance-trained athletes. Journals of Gerontology Series A: Biological Sciences & Medical Sciences, 2000. 55(11): p. B537-B544. |
| 10 | Cockcroft, E.J., et al., A single bout of high-intensity interval exercise and work-matched moderate-intensity exercise has minimal effect on glucose tolerance and insulin sensitivity in 7-to 10-year-old boys. Journal of Sports Sciences, 2018. 36(2): p. 149-155. |
| 11 | Cockcroft, E.J., et al., High intensity interval exercise is an effective alternative to moderate intensity exercise for improving glucose tolerance and insulin sensitivity in adolescent boys. Journal of Science and Medicine in Sport, 2015. 18(6): p. 720-724. |
| 12 | Fang, B., Y. Kim, and M. Choi, Effect of Cycle-Based High-Intensity Interval Training and Moderate to Moderate-Intensity Continuous Training in Adolescent Soccer Players. Healthcare (Basel), 2021. 9(12). |
| 13 | Faria, W.F., et al., Effects of 2 Methods of Combined Training on Cardiometabolic Risk Factors in Adolescents: a Randomized Controlled Trial. Pediatric exercise science, 2020. 32(4): p. 217‐226. |
| 14 | Gavanda, S., et al., The Effects of High-Intensity Functional Training Compared with Traditional Strength or Endurance Training on Physical Performance in Adolescents: A Randomized Controlled Trial. Journal of Strength & Conditioning Research, 2022. 36(3): p. 624-632. |
| 15 | Helén, J., et al., High-Intensity Functional Training Induces Superior Training Adaptations Compared With Traditional Military Physical Training. Journal of strength and conditioning research, 2023. 37(12): p. 2477-2483. |
| 16 | Jeppesen, J.S., et al., Four Weeks of Intensified Training Enhances On-Ice Intermittent Exercise Performance and Increases Maximal Oxygen Consumption of Youth National-Team Ice Hockey Players. International Journal of Sports Physiology and Performance, 2022. 17(10): p. 1507-1515. |
| 17 | Ketelhut, S., T. Kirchenberger, and R.G. Ketelhut, Hemodynamics in young athletes following high-intensity interval or moderate-intensity continuous training. J Sports Med Phys Fitness, 2020. 60(9): p. 1202-1208. |
| 18 | Leal, J.M. and F.B. Del Vecchio, Postexercise hypotension in men with parental history of hypertension: effects of mode and intensity. Journal of sports medicine and physical fitness, 2022. 62(2): p. 273‐279. |
| 19 | McManus, A.M., N.R. Sletten, and D.J. Green, The Impact of Different Exercise Intensities on Vasodilation and Shear Rate Patterns in Children. Pediatric exercise science, 2019. 31(3): p. 282-289. |
| 20 | Mendonca, F.R., et al., Effects of aerobic exercise combined with resistance training on health-related physical fitness in adolescents: A randomized controlled trial. Journal of Exercise Science & Fitness, 2022. 20(2): p. 182-189. |
| 21 | Oliveira, R., et al., Mechanisms of blood pressure control following acute exercise in adolescents: Effects of exercise intensity on haemodynamics and baroreflex sensitivity. Exp Physiol, 2018. 103(8): p. 1056-1066. |
| 22 | Ramírez-Vélez, R., et al., Similar cardiometabolic effects of high- and moderate-intensity training among apparently healthy inactive adults: a randomized clinical trial. Journal of translational medicine, 2017. 15(1): p. 118. |
| 23 | Schöffl, I., et al., Jumping into a Healthier Future: Trampolining for Increasing Physical Activity in Children. Sports Medicine - Open, 2021. 7(1): p. 1-7. |
| 24 | Sommer Jeppesen, J., et al., Four Weeks of Intensified Training Enhances On-Ice Intermittent Exercise Performance and Increases Maximal Oxygen Consumption of Youth National-Team Ice Hockey Players. Int J Sports Physiol Perform, 2022. 17(10): p. 1507-1515. |
| 25 | Su, W., et al., Distinct lipidomic profiles but similar improvements in aerobic capacity following sprint interval training versus moderate-intensity continuous training in male adolescents. Frontiers in Physiology, 2025. 16. |
| 26 | Sun, F., et al., Effect of eight-week high-intensity interval training versus moderate-intensity continuous training programme on body composition, cardiometabolic risk factors in sedentary adolescents. Front Physiol, 2024. 15: p. 1450341. |
| 27 | Thum, J.S., et al., High-intensity interval training elicits higher enjoyment than moderate intensity continuous exercise. PloS one, 2017. 12(1): p. e0166299. |
| 28 | Tolfrey, K., J.K. Zakrzewski-Fruer, and A.E. Thackray, Metabolism and Exercise During Youth-The Year That Was 2017. Pediatr Exerc Sci, 2018. 30(1): p. 38-41. |
| 29 | Umutlu, G., et al., Neuromuscular, hormonal and cardiovascular adaptations to eight-week HIIT and continuous aerobic training combined with neuromuscular electrical stimulation. Journal of sports medicine and physical fitness, 2020. 60(4): p. 510‐519. |
| 30 | van Biljon, A., et al., Do Short-Term Exercise Interventions Improve Cardiometabolic Risk Factors in Children? J Pediatr, 2018. 203: p. 325-329. |
| 31 | Van Biljon, A., et al., Short-Term High-Intensity Interval Training is Superior to Moderate-Intensity Continuous Training in Improving Cardiac Autonomic Function in Children. Cardiology, 2018. 141(1): p. 1‐8. |
| 32 | Zhang, G.F., Y. Kim, and J.Y. Lee, Impact of short term high-intensity interval training on the aerobic and anaerobic fitness of young male football players in the final stages of rehabilitation. Journal of Mens Health, 2024. 20(6): p. 108-115. |


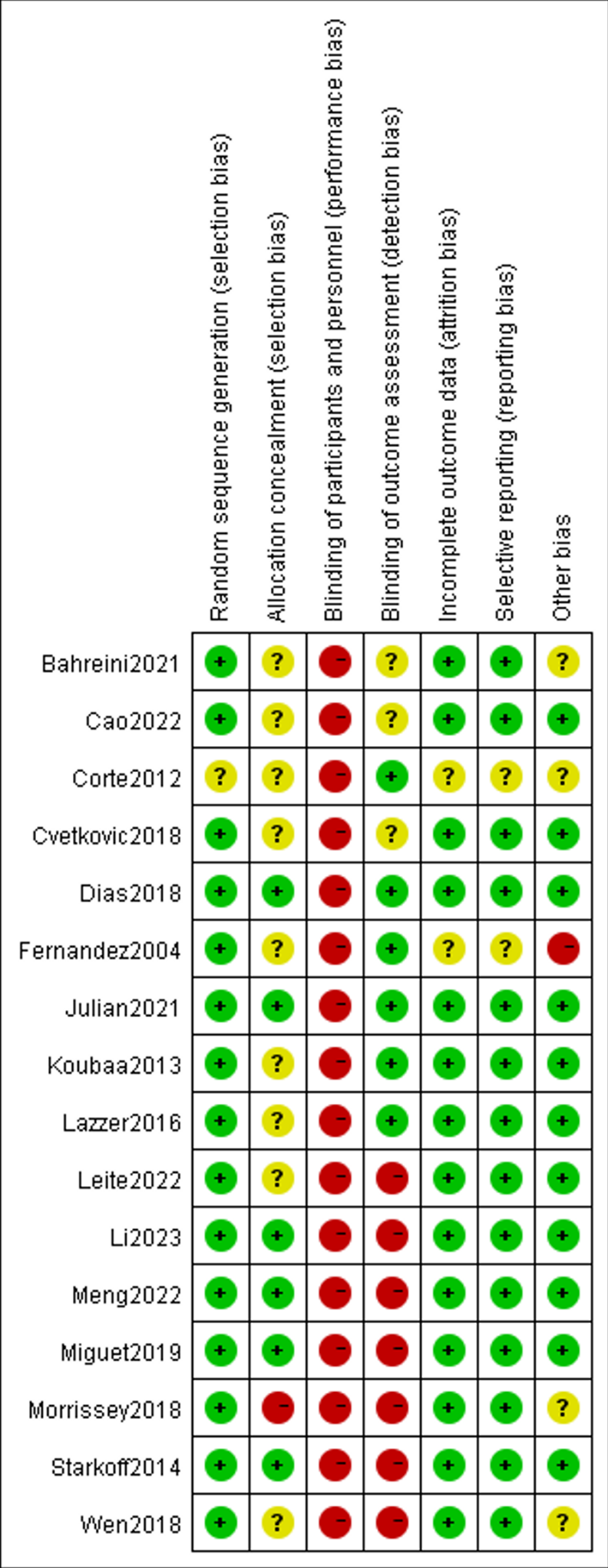


**Figure S1 Risk of bias assessment.**

**Table S9. PRISMA Abstracts checklist**

| **Section and Topic** | **Item #** | **Checklist item** | **Reported (Yes/No)** |
| --- | --- | --- | --- |
| **TITLE** | | |  |
| Title | 1 | Identify the report as a systematic review. | Yes |
| **BACKGROUND** | | |  |
| Objectives | 2 | Provide an explicit statement of the main objective(s) or question(s) the review addresses. | Yes |
| **METHODS** | | |  |
| Eligibility criteria | 3 | Specify the inclusion and exclusion criteria for the review. | Yes |
| Information sources | 4 | Specify the information sources (e.g. databases, registers) used to identify studies and the date when each was last searched. | Yes |
| Risk of bias | 5 | Specify the methods used to assess risk of bias in the included studies. | No |
| Synthesis of results | 6 | Specify the methods used to present and synthesise results. | Yes |
| **RESULTS** | | |  |
| Included studies | 7 | Give the total number of included studies and participants and summarise relevant characteristics of studies. | Yes |
| Synthesis of results | 8 | Present results for main outcomes, preferably indicating the number of included studies and participants for each. If meta-analysis was done, report the summary estimate and confidence/credible interval. If comparing groups, indicate the direction of the effect (i.e. which group is favoured). | Yes |
| **DISCUSSION** | | |  |
| Limitations of evidence | 9 | Provide a brief summary of the limitations of the evidence included in the review (e.g. study risk of bias, inconsistency and imprecision). | No |
| Interpretation | 10 | Provide a general interpretation of the results and important implications. | Yes |
| **OTHER** | | |  |
| Funding | 11 | Specify the primary source of funding for the review. | No |
| Registration | 12 | Provide the register name and registration number. | Yes |

**Table S10. PRISMA checklist.**

| **Section and Topic** | **Item #** | **Checklist item** | **Location where item is reported** |
| --- | --- | --- | --- |
| **TITLE** | | |  |
| Title | 1 | Identify the report as a systematic review. | P.1 |
| **ABSTRACT** | | |  |
| Abstract | 2 | See the PRISMA 2020 for Abstracts checklist. | P.1 |
| **INTRODUCTION** | | |  |
| Rationale | 3 | Describe the rationale for the review in the context of existing knowledge. | P.2-3 |
| Objectives | 4 | Provide an explicit statement of the objective(s) or question(s) the review addresses. | P.2-3 |
| **METHODS** | | |  |
| Eligibility criteria | 5 | Specify the inclusion and exclusion criteria for the review and how studies were grouped for the syntheses. | P.3-4 |
| Information sources | 6 | Specify all databases, registers, websites, organisations, reference lists and other sources searched or consulted to identify studies. Specify the date when each source was last searched or consulted. | P.3 |
| Search strategy | 7 | Present the full search strategies for all databases, registers and websites, including any filters and limits used. | Table S1-S6  (Supplementary material) |
| Selection process | 8 | Specify the methods used to decide whether a study met the inclusion criteria of the review, including how many reviewers screened each record and each report retrieved, whether they worked independently, and if applicable, details of automation tools used in the process. | P.4-5 |
| Data collection process | 9 | Specify the methods used to collect data from reports, including how many reviewers collected data from each report, whether they worked independently, any processes for obtaining or confirming data from study investigators, and if applicable, details of automation tools used in the process. | P.4-5 |
| Data items | 10a | List and define all outcomes for which data were sought. Specify whether all results that were compatible with each outcome domain in each study were sought (e.g. for all measures, time points, analyses), and if not, the methods used to decide which results to collect. | P.3-5 |
|  | 10b | List and define all other variables for which data were sought (e.g. participant and intervention characteristics, funding sources). Describe any assumptions made about any missing or unclear information. | P.3-5 |
| Study risk of bias assessment | 11 | Specify the methods used to assess risk of bias in the included studies, including details of the tool(s) used, how many reviewers assessed each study and whether they worked independently, and if applicable, details of automation tools used in the process. | P.6 |
| Effect measures | 12 | Specify for each outcome the effect measure(s) (e.g. risk ratio, mean difference) used in the synthesis or presentation of results. | P.5-6 |
| Synthesis methods | 13a | Describe the processes used to decide which studies were eligible for each synthesis (e.g. tabulating the study intervention characteristics and comparing against the planned groups for each synthesis (item #5)). | P.5-6 |
|  | 13b | Describe any methods required to prepare the data for presentation or synthesis, such as handling of missing summary statistics, or data conversions. | P.5 |
|  | 13c | Describe any methods used to tabulate or visually display results of individual studies and syntheses. | P.5-6 |
|  | 13d | Describe any methods used to synthesize results and provide a rationale for the choice(s). If meta-analysis was performed, describe the model(s), method(s) to identify the presence and extent of statistical heterogeneity, and software package(s) used. | P.5 |
|  | 13e | Describe any methods used to explore possible causes of heterogeneity among study results (e.g. subgroup analysis, meta-regression). | P.5 |
|  | 13f | Describe any sensitivity analyses conducted to assess robustness of the synthesized results. | P.5 |
| Reporting bias assessment | 14 | Describe any methods used to assess risk of bias due to missing results in a synthesis (arising from reporting biases). | P.5-6 |
| Certainty assessment | 15 | Describe any methods used to assess certainty (or confidence) in the body of evidence for an outcome. | P.5-6 |
| **RESULTS** | | |  |
| Study selection | 16a | Describe the results of the search and selection process, from the number of records identified in the search to the number of studies included in the review, ideally using a flow diagram. | P.5 |
|  | 16b | Cite studies that might appear to meet the inclusion criteria, but which were excluded, and explain why they were excluded. | Table S7 (Supplementary material) |
| Study characteristics | 17 | Cite each included study and present its characteristics. | P.5-6 |
| Risk of bias in studies | 18 | Present assessments of risk of bias for each included study. | P.6 |
| Results of individual studies | 19 | For all outcomes, present, for each study: (a) summary statistics for each group (where appropriate) and (b) an effect estimate and its precision (e.g. confidence/credible interval), ideally using structured tables or plots. | P.6-8 |
| Results of syntheses | 20a | For each synthesis, briefly summarize the characteristics and risk of bias among contributing studies. | P.6-8 |
|  | 20b | Present results of all statistical syntheses conducted. If meta-analysis was done, present for each the summary estimate and its precision (e.g. confidence/credible interval) and measures of statistical heterogeneity. If comparing groups, describe the direction of the effect. | P.6-8 |
|  | 20c | Present results of all investigations of possible causes of heterogeneity among study results. | P.6-8 |
|  | 20d | Present results of all sensitivity analyses conducted to assess the robustness of the synthesized results. | P.6-8 |
| Reporting biases | 21 | Present assessments of risk of bias due to missing results (arising from reporting biases) for each synthesis assessed. | P.6 |
| Certainty of evidence | 22 | Present assessments of certainty (or confidence) in the body of evidence for each outcome assessed. | P.6-8 |
| **DISCUSSION** | | |  |
| Discussion | 23a | Provide a general interpretation of the results in the context of other evidence. | P.8-12 |
|  | 23b | Discuss any limitations of the evidence included in the review. | P.13 |
|  | 23c | Discuss any limitations of the review processes used. | P.13 |
|  | 23d | Discuss implications of the results for practice, policy, and future research. | P.13 |
| **OTHER INFORMATION** | | |  |
| Registration and protocol | 24a | Provide registration information for the review, including register name and registration number, or state that the review was not registered. | P.3 |
|  | 24b | Indicate where the review protocol can be accessed, or state that a protocol was not prepared. | P.3 |
|  | 24c | Describe and explain any amendments to information provided at registration or in the protocol. | Not applicable |
| Support | 25 | Describe sources of financial or non-financial support for the review, and the role of the funders or sponsors in the review. | Not applicable |
| Competing interests | 26 | Declare any competing interests of review authors. | P.20 |
| Availability of data, code and other materials | 27 | Report which of the following are publicly available and where they can be found: template data collection forms; data extracted from included studies; data used for all analyses; analytic code; any other materials used in the review. | P.20 |

**
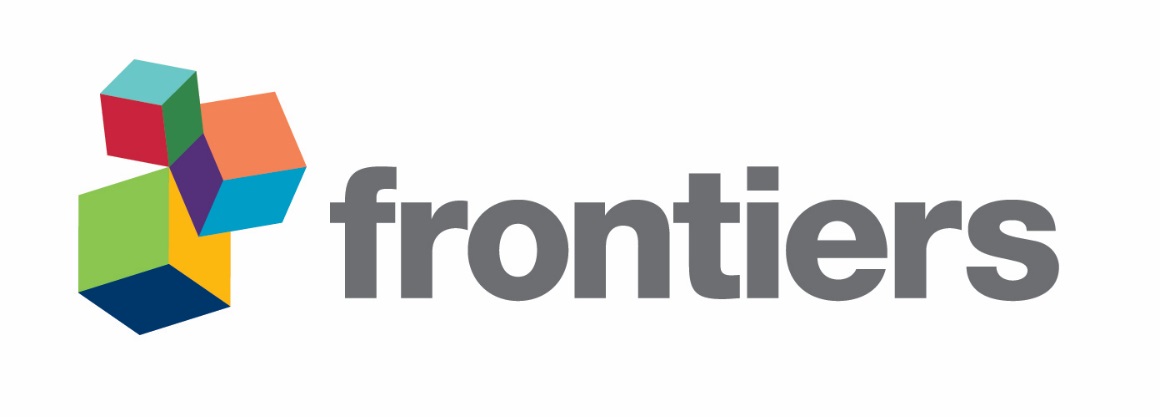
**
